# Lysosome and plasma membrane Piezo channels of *Trypanosoma cruzi* are essential for proliferation, differentiation and infectivity

**DOI:** 10.1371/journal.ppat.1013105

**Published:** 2025-04-23

**Authors:** Guozhong Huang, Mayara S. Bertolini, Justin Wiedeman, Ronald D. Etheridge, Teresa Cruz-Bustos, Roberto Docampo

**Affiliations:** 1 Center for Tropical and Emerging Global Diseases, University of Georgia, Athens, Georgia, United States of America; 2 Department of Cellular Biology, University of Georgia, Athens, Georgia, United States of America; London School of Hygiene and Tropical Medicine, UNITED KINGDOM OF GREAT BRITAIN AND NORTHERN IRELAND

## Abstract

*Trypanosoma cruzi*, the causative agent of Chagas disease, is a parasitic protist that affects millions of people worldwide. Currently there are no fully effective drugs or vaccines available. Contact of *T. cruzi* infective forms with their host cells or with the extracellular matrix increases their intracellular Ca^2+^ concentration suggesting a mechano-transduction process. We report here that *T. cruzi* possesses two distinct mechanosensitive Piezo channels, named TcPiezo1 and TcPiezo2, with different subcellular localizations but similarly essential for normal proliferation, differentiation, and infectivity. While TcPiezo1 localizes to the plasma membrane, TcPiezo2 localizes to the lysosomes. Downregulation of *TcPiezo1* expression by a novel ligand-regulated hammerhead ribozyme (HHR) significantly inhibited Ca^2+^ entry in cells expressing a genetically encoded Ca^2+^ indicator while downregulation of *TcPiezo2* expression inhibited Ca^2+^ release from lysosomes, which are now identified as novel acidic Ca^2+^ stores in trypanosomes. The channels are activated by contact with extracellular matrix and by hypoosmotic stress. The results establish the essentiality of Piezo channels for the life cycle and Ca^2+^ homeostasis of *T. cruzi* and a novel lysosomal localization for a Piezo channel in eukaryotes.

## Author summary

Chagas disease, caused by *T. cruzi* infection, affects millions of people and its treatment is not satisfactory. Our results establish the essentiality of Piezo channels for the life cycle and Ca^2+^ homeostasis of *T. cruzi* and their potential roles as targets for chemotherapy. In addition, we demonstrate the function of these channels in Ca^2+^ entry and lysosomal Ca^2+^ release in a unicellular eukaryote.

## Introduction

Chagas disease, the leading cause of congestive heart failure in Latin America [1], is caused by infection with the unicellular eukaryote *Trypanosoma cruzi*. The disease affects 6–7 million people in the Americas. Treatment of Chagas disease is restricted to drugs with relatively high toxicity and limited efficacy while no vaccines are available [2]. Understanding the biology of this parasite in greater detail is crucial for identifying novel drug targets.

It was known for some time that contact with host cells triggers an increase in cytosolic Ca^2+^ of the infective trypomastigote stage of *T. cruzi* [3]. Blocking this Ca^2+^ increase with intracellular Ca^2+^ chelators prevented host cell invasion [3,4]. The mechanism involved in the Ca^2+^ increase upon contact of trypomastigotes with host cells was never identified but its contact-dependence suggests a mechano-transduction process. Contact with extracellular matrix (ECM), a known mechano-stimulant of Piezo channels [5] has also been shown to stimulate Ca^2+^ increase in trypomastigotes [6]. In this regard, two mechanosensitive Piezo channel paralogs, which we named TcPiezo1 and TcPiezo2, are predicted to be present in *T. cruzi* [7]. Piezo channel orthologs are present in other pathogenic protists like *Leishmania spp., Trichomonas vaginalis,* and *Entamoeba histolytica,* but are absent in apicomplexan parasites (*Toxoplasma* and *Plasmodium*) and *T. brucei* [7].

Piezo channels were discovered in 2010 [8] and have essential roles in a variety of processes in eukaryotes, like vascular function, pulmonary respiration, and signal transduction [9]. They localize to the plasma membrane [8] and some intracellular structures, such as the endoplasmic reticulum [10,11], centrosomes [12], intercellular bridge of daughter cells under cytokinesis [13], and the plant vacuole [14]. They arrange in homotrimers and their mechanical activation determines a flux of Ca^2+^, which initiate intracellular signal transduction pathways [9].

Ca^2+^ signaling in *T. cruzi* has peculiarities not found in mammalian cells. A most striking peculiarity is the localization of the inositol-1,4,5-trisphosphate (IP_3_) receptor (IP_3_R) to acidocalcisomes [15], since this critical and important channel is present in the endoplasmic reticulum (ER) of most eukaryotic cells [16]. The presence of acidocalcisomes as the main intracellular Ca^2+^ store [17,18] is also unique. The phosphoinositide phospholipase C 1 (PI-PLC1), which hydrolyzes phosphatidyl inositol 4,5-bisphosphate (PIP_2_) to IP_3_ [19], is N-terminally myristoylated and palmitoylated and stimulated by Ca^2+^ [20,21]. The SERCA-Ca^2+^-ATPase, the Ca^2+^ pump in the ER that maintains cytosolic Ca^2+^ levels low [22], is thapsigargin-insensitive [23]. The organization of the mitochondrial Ca^2+^ uniporter (MCU) complex is also peculiar, with additional subunits and missing ones [24–26]. All these peculiarities point to differences in the Ca^2+^ signaling pathways that trigger significant steps of the virulence routes involved in *T. cruzi* parasitism.

Ca^2+^ signaling pathways regulate critical cellular processes of *T. cruzi* such as differentiation [27], host cell invasion [3], osmoregulation [28], and cell bioenergetics [29]. However, elucidating the role of essential genes in those processes has been hampered by the lack of a conditional gene knockdown system and a reliable Ca^2+^ indicator. The parasite does not have individual promoters for almost all its genes and it does not have RNAi machinery, which is present in *T. brucei* [30]. There are also limitations to the use of chemical Ca^2+^ indicators such as Fura-2/AM, which compartmentalize or has inefficient cleavage of its acetoxymethyl ester (AM) in the epimastigote stage. Genetically encoded Ca^2+^ indicators (GECIs) overcome these limitations.

In this work we report that TcPiezo1 localizes to the plasma membrane while TcPiezo2 surprisingly localizes to the lysosomes. A newly developed conditional knockout system (also called as Small Hammerhead Aptazyme-Regulated Knockdown or SHARK) for *T. cruzi* [31] and a high-performance GECI [32] are, for the first time, used to define the critical role of these channels in the parasite. Both channels are mechanosensitive, essential for normal growth, differentiation, and infectivity of *T. cruzi,* and are directly involved in Ca^2+^ signaling.

## Results

### Characterization of Piezo channels in *T. cruzi*

Two phylogenetically distinct genes are present in *T. cruzi* (Y C6 strain): *TcPiezo1* (TcYC6_0088320, KAF8295942) in chromosome 7 and *TcPiezo2* (TcYC6_0007880, KAF8281887) in chromosome 5, encoding proteins with 39 and 41 transmembrane domains (TMD), respectively, and containing the PFEW motif found in other Piezo channels [7]. The ORFs predict 2606- and 2514-amino acid-proteins with apparent molecular weights of 296 and 280 kDa, respectively, which share 20.4% identity and 35.3% similarity. Both TcPiezo1 and TcPiezo2 have typical Piezo features: a divergent N-terminal mechanotransduction module and a conserved C-terminal pore region. Protein sequence alignment of C-terminal pore regions of *T. cruzi* Piezo proteins with mammalian Piezo channels (S1 Fig) reveals that TcPiezo1 and 2 contain six conserved hydrophobic domains (TM33-TM38) and a glutamate residue (E2133) that are essential for ion (Ca^2+^) selectivity and pore blocking [33]. Recently, the CryoEM structures of mouse Piezo channels (mPiezo1 and 2) became available [34–36], but the low sequence identity between mPiezo and TcPiezo proteins (<19%) renders it unfeasible to perform conventional template-based homology modeling. We used AlphaFold II [37] to predict the structures of the Piezo conserved C-terminal sequences, as aligned in S1 Fig. As expected, the structures of mPiezo1 and mPiezo2 predicted by AlphaFold II (S2 Fig) resemble those obtained by CryoEM results [34–36]. Strikingly, the structures of TcPiezo1 and TcPiezo2 are very similar to those of mPiezo1 and mPiezo2 (S2 Fig), such as the critical E2133 residue located at the anchor and the inner helix-CTD region formed the transmembrane gate and cytosolic constriction neck of the ion (Ca^2+^) pathway [35].

### Attempts to knockout (KO) *TcPiezo1* and *TcPiezo2* gene expression

We designed a CRISPR/Cas9 strategy (S3A Fig) to generate KO mutants of the pore region or whole *TcPiezo1* and *TcPiezo2* genes. The method involves the constitutive expression of Cas9 and specific single guide RNA (sgRNA) and the utilization of a DNA donor template to promote double-strand break repair by homologous-directed repair [38]. Three cassettes were utilized along with two unique *TcPiezo1*- or *TcPiezo2*- targeted sgRNA designs. Each of the two unique designs was used in technical duplicates and the complete co-transfection experiments were repeated a total of three independent times. Multiple CRISPR/Cas9 genome editing experiments to knock out the pore region or the whole *TcPiezo1* and *TcPiezo2* genes were attempted by using different sgRNAs with blasticidin (*Bsd)* or puromycin (*Puro*) resistance gene cassettes, but no epimastigotes survived the selection process with minimal concentration of these antibiotics for *T. cruzi*, suggesting the essentiality of these genes for cell growth.

### Conditional knockout of *TcPiezo1* and subcellular localization

We then used a newly developed conditional knockout method based on the tetracycline or theophylline-dependent activation of a hammerhead ribozyme (S3F Fig), recently described (Shark 1 and Shark3, respectively) [31].

**Fig 1:**
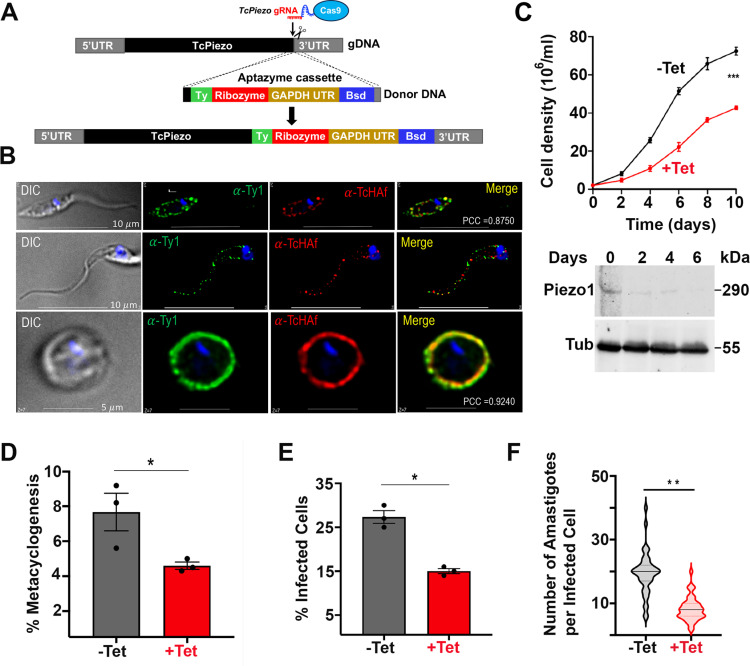
Conditional knockout of *TcPiezo1* and subcellular localization. (A) Schematic representation of CRISPR/Cas9 mediated endogenous C-terminal tagging of *TcPiezo1* with an aptazyme cassette Shark1 or Shark3 (composed of 3 × Ty tag, ribozyme, GAPDH 3’UTR, and *Bsd* gene) for conditional knockout (CKO) of *TcPiezo1*. (B) Ty1-tagged TcPiezo1 Tet-OFF colocalization with *T. cruzi* plasma membrane marker H^+^-ATPase (TcHAf or α-HAf) in the plasma membrane of epimastigote (Pearson’s correlation coefficient (PCC): 0.8750), trypomastigote (PCC: 0.6021) and amastigote (PCC: 0.9240), as detected by immunofluorescence analyses with antibodies against Ty1. Yellow in merged images indicates colocalization. DIC, differential interference contrast microscopy. DAPI staining in blue. Scale bars 5 and 10 µm, as indicated. (C) Growth of *TcPiezo1 Tet-OFF* epimastigotes in the absence (black line, *− Tet*) or presence (red line, *+ Tet*) of 5 μg/ml tetracycline for the indicated number of days. Western blot analyses of *TcPiezo1 Tet-OFF* epimastigotes grown in the absence (0) or presence (2–6) of tetracycline. Total lysates (30 μg) were subjected to 10% SDS-polyacrylamide gel electrophoresis before transfer to a nitrocellulose membrane and then stained with antibodies against Ty1 (top). One band of ~ 290 kDa was detected in epimastigote homogenates. Membranes were stripped and re-incubated with antibody against Alpha-tubulin as a loading control (bottom, Tub). (D) Percentage (%) of metacyclic trypomastigotes in *TcPiezo1 Tet-OFF* epimastigote cultures after incubation in TAU 3AAG medium in the absence (-Tet) or presence (+Tet) of 5 µ g/ml tetracycline after 96 h. Differentiation of epimastigotes to metacyclic trypomastigotes was quantified by staining with DAPI to distinguish the position of the kinetoplast by fluorescence microscopy. (E) Effect of non-induced (-Tet) and induced (+Tet) *TcPiezo1 Tet-OFF* on trypomastigote infection of Vero cells after 4 h. (F) Effect of non-induced (-Tet) and induced (+Tet) *TcPiezo1 Tet-OFF* on amastigote replication after 72 h. In panels C, D, E, F, values are means ± s.d. (*n* = 3). One-way ANOVA with multiple comparisons (*P < 0.05, **P < 0.01, ***P < 0.001).

In this system, we used the CRISPR/Cas9-mediated endogenous C-terminal tagging of *T. cruzi* epimastigotes [15] to introduce a *Ty1* tag sequence into the *TcPiezo1* gene, followed by the tetracycline/theophylline inducible ribozyme, a blasticidin deaminase (*Bsd*) gene and the *TcPiezo1* 3’UTR (Figs 1A and S3B–D). Fig 1B, top panels, is the immunofluorescence analysis (IFA) of TcPiezo1-Ty1-labeled epimastigotes showing punctate labeling that co-localizes with a plasma membrane marker (antibody TcHAf against a *T. cruzi* P-type H^+^-ATPase (α-HAf) [39]). To investigate the localization of TcPiezo1 in the infective stages we induced the differentiation of TcPiezo1-Ty1 epimastigotes into infective metacyclic trypomastigotes by incubating them in triatome artificial urine (TAU) medium as described previously [40]. We then infected Vero cells, collected the infective stages (trypomastigotes and amastigotes), and performed IFAs. Fig 1B, central panel, shows a surface punctate localization of TcPiezo1-Ty1 in trypomastigotes that does not perfectly coincide with the punctate surface localization of TcHAf. Fig 1B, lower panel, shows that TcPiezo1-Ty1 co-localizes with anti-TcHAf to the plasma membrane of amastigotes. Labelling of amastigotes was stronger and more continuous. When analyzed by western blot using antibodies against Ty1, a clear band of ~ 290 kDa (predicted size) was detected in the TcPiezo1-Ty1 cell lysates of two clones each of Tet-OFF and Theo-OFF epimastigotes (S4A Fig). The signal was weak because of the low expression levels of this protein. Knockdown of *TcPiezo1,* induced by addition of tetracycline, resulted in a marked growth defect in epimastigotes from day 4, with a correlative decrease in *TcPiezo1* expression, as analyzed by western blot (Fig 1C). Similar results were observed when knockdown was induced by theophylline addition to epimastigotes (S4B Fig). Therefore, all further phenotypic analyses were conducted on day 4 of tetracycline or theophylline induction, unless indicated otherwise.

Knockdown of *TcPiezo1* expression by addition of tetracycline (Figs 1D–F and S4F) or theophylline (S4C–E and S4G Fig) significantly inhibited metacyclogenesis, tissue culture-derived trypomastigote invasion of host cells and amastigote replication within Vero cells as compared with Tet-OFF or Theo-OFF cells. It has been reported before that there is no difference in the proliferation rate of wild type cells after treatment with Tet or Theo [31].

These results demonstrate that TcPiezo1 localizes to the plasma membrane of *T. cruzi* and is essential for its normal proliferation, differentiation and infectivity.

### Expression of jGCaMP7s in *TcPiezo1* transgenic cell lines

To investigate the effects of downregulation of *TcPiezo1* and of Piezo activators and inhibitors on Ca^2+^ entry, we initially used Fura-2/AM loaded cells. However, although this method could be used with amastigotes to show inhibition of Ca^2+^ entry by downregulation of *TcPiezo1* expression (S5A, B Fig), loading of epimastigotes resulted in inefficient esterase-catalyzed cleavage of the acetoxymethyl ester. We therefore expressed genetically encoded Ca^2+^ indicators (GECIs) in *TcPiezo1* transgenic cell lines. GCaMP6 and jGCaMP7 are high performance sensitive GECIs with slow (s) and fast (f) Ca^2+^ binding kinetics in neurons [41,42]. To determine the sensitivity of those GCaMP variants to Ca^2+^ changes in *T. cruzi*, we transfected WT epimastigotes with GCaMP6s, GCaMP6f, jGCaMP7s and jGCaMP7f (S6A Fig), isolated cells with low fluorescence using cell sorting, and analyzed them for their response to ionomycin (S6B Fig). jGCaMP7s transfected cells gave maximal fluorescence response to the ionophore (S6B Fig). These cells were labeled by immunofluorescence analysis (IFA) with anti-GFP (S6C Fig) and showed bands of 50 kDa by western blot analyses (S6A Fig). Based on those results, we generated transgenic *TcPiezo1 Tet-OFF/Theo-OFF* cell lines expressing jGCaMP7s, which were verified by IFA and western blot analyses with anti-His antibody (S6D, E Fig). The *TcPiezo1-CKO* cell lines expressing jGCaMP7s released similar amounts of intracellular Ca^2+^ when either Tet-OFF or Theo-OFF cells were exposed to ionomycin (S6F Fig). We used them in our experiments.

**Fig 2:**
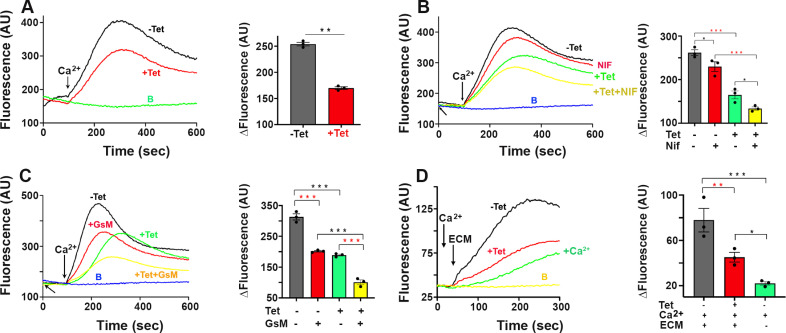
Downregulation of *TcPiezo1* expression decreases Ca^2+^ entry in *T. cruzi.* Intracellular Ca^2+^ concentrations of 5 × 10^7^ *TcPiezo1 Tet-OFF* epimastigotes (A-C) or 2 x 10^7^ trypomastigotes (D) expressing jGCaMP7s were measured by jGCaMP7s signal fluorescence in arbitrary units of fluorescence (AU). An increase in jGCaMP7s fluorescence indicates increasing cytosolic Ca^2+^. (A) Addition of 1.8 mM Ca^2+^ in Tet-induced *TcPiezo1* cells (+Tet) elicited a lower increase in intracellular Ca^2+^ than in non-induced cells (-Tet). (B) Downregulation of *TcPiezo1* expression showed a significant decrease of intracellular Ca^2+^ (+Tet). Addition of 10 µ M nifedipine (the TcCa_v_ inhibitor) slightly reduced intracellular Ca^2+^ in non-induced cells (-Tet + Nif) while it had an additive effect in Tet-induced cells (+Tet + Nif). (C) Addition of 10 µ M GsMTx4 showed a significant reduction of intracellular Ca^2+^ in non-induced cells (-Tet + GsM) comparable to downregulation of *TcPiezo1* (+Tet) and had an additive effect in Tet-induced cells (+Tet + GsM). (D) Extracellular matrix (ECM) triggered TcPiezo1-mediated Ca^2+^ entry in tissue culture-derived trypomastigotes. Addition of 40 µg ECM to non-induced (-Tet) trypomastigotes significantly increased intracellular Ca^2+^, compared to Tet-induced cells (+Tet). In panels A-D, addition of buffer A with glucose (BAG), instead of 1.8 mM Ca^2+^, was used as a control baseline labeled with B. Values are means ± s.d. (n = 3). One-way ANOVA with multiple comparisons (*P < 0.05, **P < 0.01, ***P < 0.001).

Fig 2A shows that addition of 1.8 mM CaCl_2_ to *TcPiezo1-CKO* transgenic epimastigotes expressing jGCaMP7s resulted in an increase in intracellular Ca^2+^, which was significantly reduced when tetracycline was added to downregulate *TcPiezo1* expression. Similar results were obtained upon addition of theophylline to downregulate *TcPiezo1* expression (S5C Fig).

Addition of nifedipine, an inhibitor of the voltage-dependent Ca^2+^ channel (TcCa_V_) on the surface of *T. cruzi* [43], enhances the inhibitory effect of *TcPiezo1* downregulation (Fig 2B). This suggests that both TcPiezo 1 and TcCa_V_ channels are involved in Ca^2+^ entry. In addition, the Piezo inhibitor GsMTx4 has an inhibitory effect equivalent to the expression downregulation of *TcPiezo1* (Fig 2C).

In agreement with previous reports on the effect of extracellular matrix (ECM) on Ca^2+^ increase in trypomastigotes [6], addition of ECM in the presence of extracellular Ca^2+^ resulted in Ca^2+^ increase that was greatly reduced in trypomastigotes in which *TcPiezo1* expression was downregulated by addition of either tetracycline (Fig 2D) or theophylline (S5D Fig).

These results indicate that TcPiezo1 mediates Ca^2+^ entry in *T. cruzi,* which is inhibited by GsMTx4 and stimulated by the mechano-stimulant ECM.

### Subcellular localization of TcPiezo2 to lysosomes

**Fig 3:**
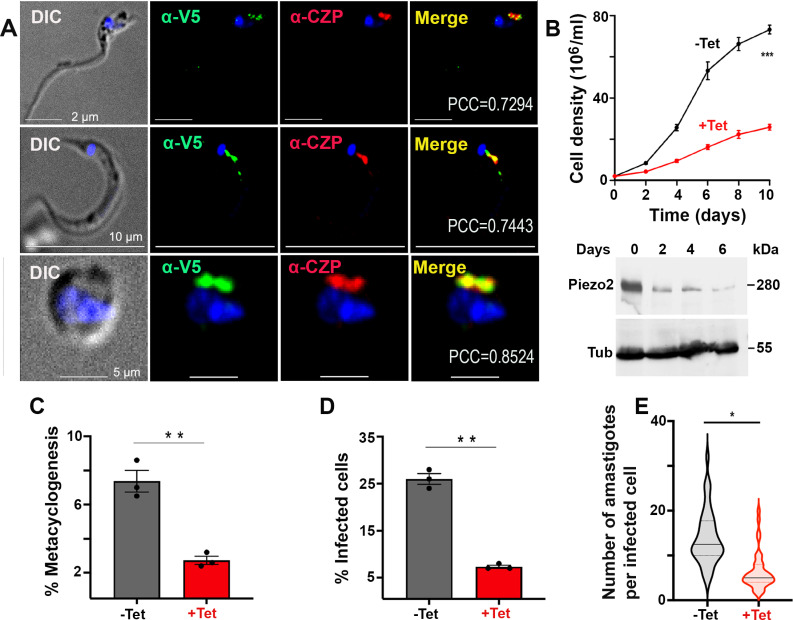
Subcellular localization and conditional knockout (*CKO*) of *TcPiezo2.* (A) Endogenous C-terminally smV5-tagged TcPiezo2 colocalized with *T. cruzi* cruzipain (TcCZP) to the reservosomes/lysosomes of epimastigotes (PCC: 0.7294), trypomastigotes (PCC: 0.7443) and amastigotes (PCC: 0.8524). Yellow in merged images indicates colocalization. DIC, differential interference contrast microscopy. DAPI staining in blue. Scale bars 5 and 10 µm, as indicated. (B) Growth of *TcPiezo2 Tet-OFF* epimastigotes in the absence (black line, *− Tet*) or presence (red line, *+ Tet*) of 5 μg/ml tetracycline for the indicated number of days. Western blot analyses of *TcPiezo2 Tet-OFF* epimastigotes grown in the absence (0) or presence (2–6) of tetracycline. Total lysates (30 μg) were subjected to 10% SDS-polyacrylamide gel electrophoresis before transfer to a nitrocellulose membrane and then stained with antibodies against Ty1 (top). One band of ~ 280 kDa was detected in epimastigote homogenates. Membranes were stripped and re-incubated with antibody against Alpha-tubulin as a loading control (bottom, Tub). (C) Percentage of metacyclic trypomastigotes in *TcPiezo2 Tet-OFF* epimastigote cultures after incubation in TAU 3AAG medium in the absence (-Tet) and presence (+Tet) of 5 µ g/ml tetracycline after 96 h. (D) Effect of non-induced (-Tet) and induced (+Tet) *TcPiezo2 Tet-OFF* on trypomastigote infection of Vero cells after 4 h. (E) Effect of non-induced (-Tet) and induced (+Tet) *TcPiezo2 Tet-OFF* on amastigote replication after 72 h. In panels B, C, D, E, values are means ± s.d. (*n* = 3). One-way ANOVA with multiple comparisons (*P < 0.05, **P < 0.01, ***P < 0.001).

To investigate the localization of TcPiezo2 (Figs 3A and S7A),its C-terminus was tagged in epimastigotes with a high performance “Spaghetti monster” fluorescent protein (smFP, smV5) tag using homologous recombination with the endogenous gene locus and a pMOTag2mV vector, previously modified for endogenous tagging of weakly expressed membrane proteins in *T. brucei* [44]. Western blot analysis confirmed the expression of the protein of the expected size (S7B Fig) at higher levels of expression than those of TcPiezo1 (S4A Fig). TcPiezo2 localized to the reservosomes and lysosomes, as demonstrated by colocalization with antibodies against *T. cruzi* reservosomal/lysosomal markers cruzipain [45] (Fig 3A) and serine carboxypeptidase [46] (S7A Fig). Similar colocalizations were found in trypomastigotes and amastigotes (Figs 3A and S7A). These experiments also demonstrate the utility of pMOTag2mV for endogenous C-terminus tagging in *T. cruzi* without the use of CRISPR/Cas9 genome editing. Further confirmation of this localization was obtained by IFA of epimastigotes tagged with Ty1 used for conditional knockout of *TcPiezo2* (S7I Fig), as described below.

### Conditional knockout of *TcPiezo2* and phenotypic changes

The same approach used with *TcPiezo1* (Figs 1A and S3B–F) was applied to knockdown the expression of *TcPiezo2*. Knockdown of *TcPiezo2* by addition of tetracycline resulted in a marked growth defect in epimastigotes with a correlative decrease in *TcPiezo2* expression, as analyzed by western blot (Fig 3B). Similar results were observed when knockdown was induced by theophylline addition to epimastigotes (S7C Fig). All further phenotypic analyses of TcPiezo2 transfectants were also done on day 4 of tetracycline or theophylline induction.

As occurs with *TcPiezo1*, knockdown of *TcPiezo2* expression by addition of tetracycline (Figs 3C–E and S7G) or theophylline (S7D–F and S7H Fig) significantly inhibited metacyclogenesis, trypomastigote invasion of host cells, and amastigote replication within Vero cells.

**Fig 4:**
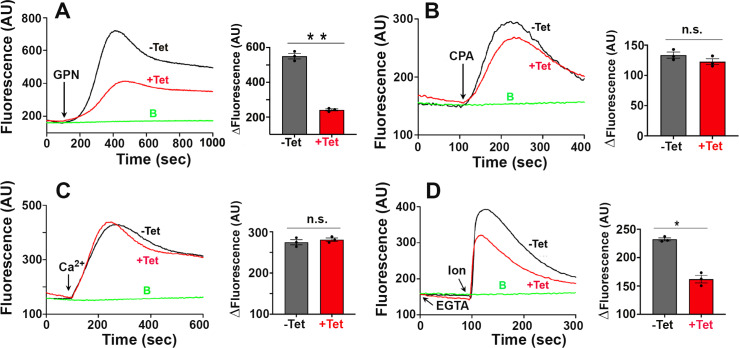
Effects of *TcPiezo2* expression downregulation on intracellular Ca^2+^ stores in *T. cruzi.* Intracellular Ca^2+^ concentrations of 5 × 10^7^ *TcPiezo2 Tet-OFF* epimastigotes expressing jGCaMP7s were measured by jGCaMP7s signal fluorescence in AU. (A) TcPiezo2 mediated lysosomal Ca^2+^ release. Addition of 70 µ M Glycyl-L-phenylalanine 2-naphthylamide (GPN) for activation of lysosomal Ca^2+^ release in Tet-induced *TcPiezo2* cells (+Tet) elicited less increase in intracellular Ca^2+^ than in non-induced cells (-Tet). (B) Addition of 30 µ M cyclopiazonic acid (CPA) for activation of ER Ca^2+^ release demonstrated no significant difference between Tet-induced (+Tet) and non-induced (-Tet) cells. (C) Addition of 1.8 mM Ca^2+^ showed no significant difference in intracellular Ca^2+^ between Tet-induced (+Tet) and non-induced (-Tet) cells. (D) Addition of 1 µ M ionomycin in Tet-induced cells (+Tet) elicited less increase of intracellular Ca^2+^ than in non-induced cells (-Tet). 100 µ M EGTA was added to remove extracellular Ca^2+^ in A-D. Addition of DMSO or BAG was used as control (baseline) labeled with B. Values are means ± s.d. (n = 3). One-way ANOVA with multiple comparisons (*P < 0.05, **P < 0.01).

To investigate the role of TcPiezo2 in Ca^2+^ homeostasis in *T. cruzi*, we then expressed jGCaMP7s in *TcPiezo2-CKO* transgenic cell lines. The cells were labeled by immunofluorescence analysis (IFA) with anti-His antibody (S6G Fig), showed bands of 50 kDa by western blot analyses (S6H Fig), and released similar amounts of intracellular Ca^2+^ when either Tet-OFF or Theo-OFF cells were exposed to ionomycin (S6I Fig). We investigated the effects of downregulation of *TcPiezo2* on intracellular Ca^2+^ stores in these epimastigotes (Fig 4). Experiments were done in the absence of extracellular Ca^2+^ (100 µ M EGTA added) to prevent Ca^2+^ influx. Fig 4A shows that addition of 70 µ M glycyl-L-phenylalanine 2-naphthylamide (GPN), a widely used lysosomotropic compound that releases Ca^2+^ from lysosomes [47], to *TcPiezo2-CKO* transgenic epimastigotes expressing jGCaMP7s, resulted in an increase in intracellular Ca^2+^. This increase was significantly reduced when tetracycline was added to downregulate *TcPiezo2* expression (Fig 4A). Similar results were obtained upon addition of theophylline to downregulate *TcPiezo2* expression (S8A Fig). In contrast, Ca^2+^ increase upon addition of cyclopiazonic acid (CPA), an inhibitor of the endoplasmic reticulum (ER) Ca^2+^-ATPase [23], was not significantly reduced when downregulation of *TcPiezo2* expression was obtained by addition of tetracycline (Fig 4B) or theophylline (S8B Fig). When 1.8 mM CaCl_2_ was added to transgenic parasites, Ca^2+^ influx occurred, and was not affected when *TcPiezo2* expression was downregulated in the presence of tetracycline (Fig 4C) or theophylline (S8C Fig). In contrast, when ionomycin was added in the absence of extracellular Ca^2+^ (100 µ M EGTA added), there was an increase in intracellular Ca^2+^ which was inhibited by downregulation of *TcPiezo2* expression by either tetracycline (Fig 4D) or theophylline (S8D Fig). Ionomycin is not thought to mobilize Ca^2+^ from acidic organelles [48] and the increase in Ca^2+^ by ionomycin in the absence of Ca^2+^ entry in the control suggest Ca^2+^ release from a non-acidic or neutral compartment, like the ER. The lower release by TcPiezo2 knockdown cells suggest that less Ca^2+^ is in this compartment. It is possible that this could indicate that TcPiezo2 Ca^2+^ release from lysosomes could have a function in refilling Ca^2+^ neutral stores in the absence of Ca^2+^ entry. Taken together these results are consistent with the intracellular localization of TcPiezo2, its involvement in Ca^2+^ release from acidic compartments and its lack of involvement in Ca^2+^ entry or in Ca^2+^ release from the ER.

### Effects of Piezo activators and inhibitors on TcPiezo channels

Yoda1 was identified by a high-throughput screening as the first selective activator of mouse and human Piezo1 with no activity on Piezo2 [49]. A search for Yoda1 analogs led to the synthesis of Dooku1, which acted as antagonist of Yoda1-induced effects without the ability to activate Piezo channels [50]. However, further work revealed that Dooku1can also activate Piezo1 in red blood cells [51]. Two additional small molecules of different structure and known as Jedi1 and Jedi2 were also shown to activate mPiezo1 [52].

**Fig 5:**
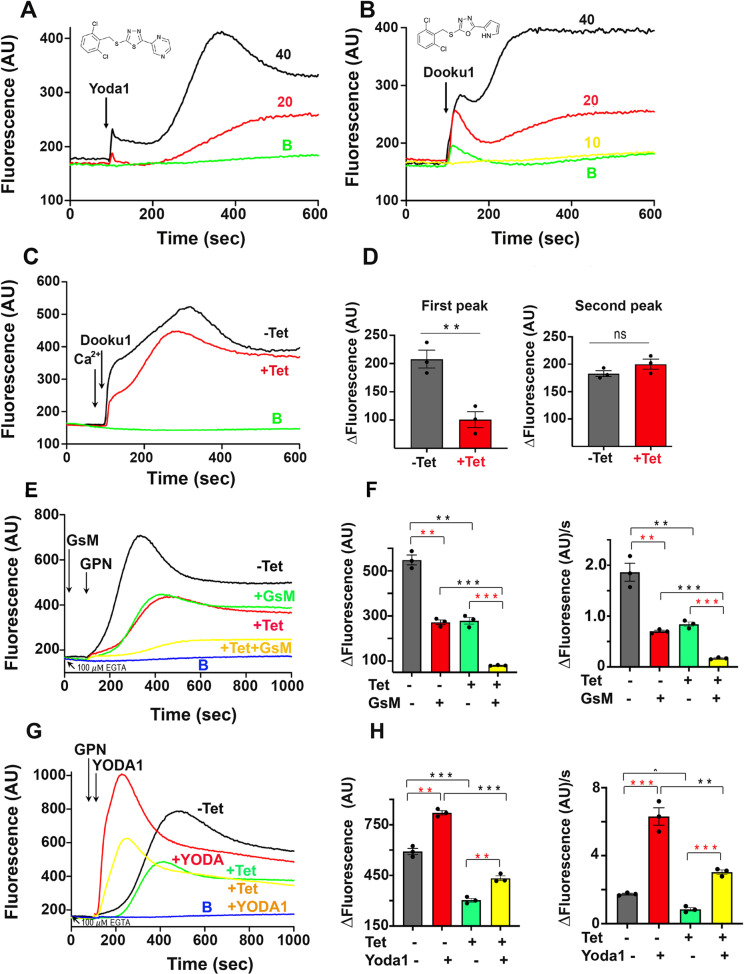
Effects of activators and inhibitors on TcPiezo channels. Intracellular Ca^2+^ changes of 5 × 10^7^ *TcPiezo Tet-OFF* epimastigotes expressing jGCaMP7s were measured by jGCaMP7s signal fluorescence in AU. (A) Yoda1 activated TcPiezo channels. Addition of 20 µ M or 40 µ M Yoda1 to *TcPiezo1 Tet-OFF* cells elicited two distinct peaks of Ca^2+^ increase, indicating that TcPiezo1 and TcPiezo2 were independently activated. (B) Addition of 20 or 40 µ M Dooku1 to the cells elicited two different peaks of Ca^2+^ rise, also suggesting that the two TcPiezo channels were successively activated. (C) Two Ca^2+^ spikes were generated in Tet-induced (+Tet) and non-induced (-Tet) *TcPiezo1 Tet-OFF* cells, respectively, by the additions of 1.8 mM Ca^2+^ and 40 µ M Dooku1. (D) Changes in jGCaMP7s fluorescence (first peak and second peak in Fig 5C) in Tet-induced (+Tet) and non-induced (-Tet) cells. Downregulation of *TcPiezo1* expression significantly decreased Dooku1-evoked TcPiezo1-mediated Ca^2+^ entry (left bar graph) but had no effect on Dooku1-evoked TcPiezo2-mediated Ca^2+^ release (right bar graph). (E) 100 µ M EGTA was incubated with *TcPiezo2 Tet-OFF* cells to remove extracellular Ca^2*+*^, abolishing TcPiezo1-mediated Ca^2+^ entry. 70 µ M GPN was added to activate Ca^2+^ release from lysosomes. Downregulation of *TcPiezo2* expression (+Tet) showed a significant decrease of lysosomal Ca^2+^ release as in Fig 4A. Addition of 10 µ M GsMTx4 showed a significant reduction of intracellular Ca^2+^ in non-induced cells (-Tet + GsM) like downregulation of *TcPiezo2* (+Tet) and had an additive effect on TcPiezo2-mediated Ca^2+^ release in Tet-induced cells (+Tet + GsM). (F) Changes in jGCaMP7s fluorescence (left bar graph) and the rates of fluorescence increase (right bar graph) upon GsM inhibition in Tet-induced (+Tet ± GsM) and non-induced (-Tet ± GsM) cells in Fig 5E. (G) 100 µ M EGTA and 70 µ M GPN were added to *TcPiezo2 Tet-OFF* cells (±Tet), as indicated. Addition of 20 µ M Yoda1 (+Yoda1) dramatically stimulated TcPiezo2-mediated Ca^2+^ release from lysosomes. (H) Changes in fluorescence (left bar graph) and the rates of fluorescence increase (right bar graph) upon Yoda1 activation in Tet-induced (+Tet±Yoda1) and non-induced (-Te±Yoda1) cells in Fig 5G. Addition of DMSO, instead of Yoda1/Dooku1/GsM, was used as control (baseline) labeled with B. In panels D, F, H, values are means ± s.d. (*n* = 3). One-way ANOVA with multiple comparisons (**P < 0.01, ***P < 0.001).

Addition of Yoda1 to transgenic epimastigotes expressing jGCaMP7s, in the presence of only contaminant Ca^2+^ (~10 µ M), resulted in a dose-dependent increase of two peaks of Ca^2+^ increase (Fig 5A). The first was tentatively attributed to Ca^2+^ entry through activation of TcPiezo1 and the second to Ca^2+^ release from an intracellular store by activation of TcPiezo2. Its analog Dooku1 has greater stimulatory activity (Fig 5B). To further demonstrate our peak assignments, we applied Dooku1 and 1.8 mM Ca^2+^ resulting in two peaks of Ca^2+^ increase (Fig 5C). Downregulation of *TcPiezo1* expression with either tetracycline (Fig 5C) or theophylline induction (S9A Fig) decreased the first peak of Ca^2+^ due to TcPiezo1 activation but did not affect the Ca^2+^ increase due to TcPiezo2 activation (Figs 5C, D and S9A). In conclusion, both Yoda1 and Dooku1 can activate TcPiezo1 and TcPiezo2.

We then tested the effect of inhibitors (GsMTx4) and activators (Yoda1) on TcPiezo2 ability to alter Ca^2+^ release by GPN from acidic intracellular stores of *T. cruzi* epimastigotes. To prevent Ca^2+^ entry we incubated the cells in the presence of 100 µ M EGTA. GsMTx4 (GsM) was able to inhibit Ca^2+^ release from acidic compartments similar to observations resulting from downregulation of *TcPiezo2* expression and both treatments together had an additive effect (+Tet, Figs 5E, 5F, + Theo, and S9B). On the other hand, Yoda1 stimulated Ca^2+^ release by GPN from acidic compartments either in control cells or in cells where *TcPiezo2* expression was downregulated (+Tet, Figs 5G and 5H, + Theo, and S9C). Interestingly, Jedi1/Jedi2 had no stimulatory activity on TcPiezo channels (S9D Fig).

These results indicate that TcPiezo-mediated Ca^2+^ fluxes in *T. cruzi*, are also able to be elicited by Yoda1 and Dooku1 instead of a mechanosensitive stimulus and are blocked by GsMTx4.

### Role of TcPiezo1 and TcPiezo2 in Ca^2+^ increase during osmotic stress

**Fig 6:**
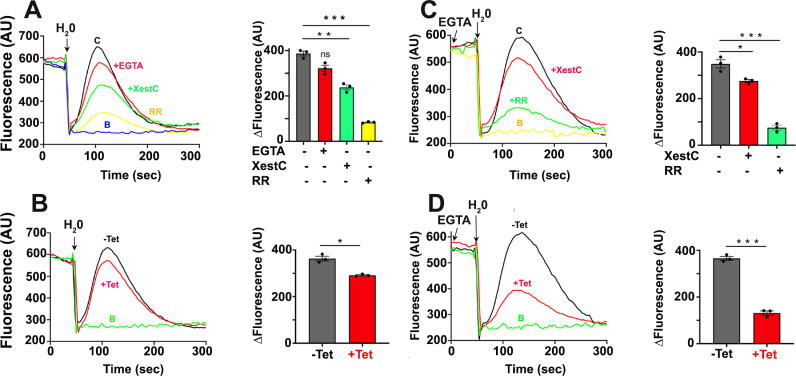
*TcPiezo-CKO* responses to hypoosmotic stress. 5 × 10^7^ *TcPiezo-CKO* epimastigotes expressing jGCaMP7s in 600 µl isosmotic buffer (300 mOsm) was submitted to hypoosmotic stress (150 mOsm) by adding 600 µl H_2_O at 50 s. Upon hypoosmotic stress, the cells swelled and the intracellular Ca^2+^ rose (control, C or -Tet). No intracellular Ca^2+^ was increased by adding 600 µl isosmotic buffer (baselines, B). (A) Non-induced *TcPiezo1 Tet-OFF* intracellular Ca^2+^ responses to the addition of 100 µ M EGTA, 1 µ M Xestospongin C (XestC), or 4 µ M ruthenium red (RR). The addition of EGTA did not affect intracellular Ca^2+^ rise, but the addition of XestC or RR did. (B) Tet-induced *TcPiezo1 Tet-OFF* cells (+Tet) had slightly lower Ca^2+^ rise than non-induced cells (-Tet). (C) Non-induced *TcPiezo2 Tet-OFF* (-Tet) intracellular Ca^2+^ responses to the addition of 1 µ M XestC or 4 µ M RR. The addition of XestC or RR significantly affected intracellular Ca^2+^ rise. (D) Tet-induced *TcPiezo2 Tet-OFF* cells (+Tet) had a significant decrease of intracellular Ca^2+^ rise, compared to non-induced cells (-Tet). 100 µ M EGTA was added to remove extracellular Ca^2*+*^, abolishing Ca^2+^ entry, as indicated. Values are means ± s.d. (*n* = 3). One-way ANOVA with multiple comparisons (*P < 0.05, **P < 0.01, *** *P* < 0.001).

Previous studies reported an increase in intracellular Ca^2+^ in different *T. cruzi* stages during hypoosmotic stress caused by 50% reduction in osmolarity from 300 to 150 mOsm [28]. This Ca^2+^ increase was attributed to Ca^2+^ entry through the plasma membrane since it was prevented by incubation with 1 mM extracellular EGTA [28]. To investigate the role of TcPiezo1 in this Ca^2+^ increase we incubated *TcPiezo1 Tet-OFF* epimastigotes expressing jGCaMP7s with 100 µ M extracellular EGTA (Fig 6A) to abolish Ca^2+^ entry, but this treatment did not significantly affect the Ca^2+^ rise (Fig 6A). However, downregulation of *TcPiezo1* expression resulted in a slight but significant decrease in intracellular Ca^2+^ rise (Fig 6B). Intracellular Ca^2+^ rise was more dramatically affected by treatment with the cell-permeable inositol 1,4,5-trisphosphate receptor (IP_3_R) inhibitor xestospongin C or by the general Ca^2+^ channel inhibitor ruthenium red (Fig 6A). To investigate the role of TcPiezo2 we incubated *TcPiezo2-OFF* epimastigotes expressing jGCaMP7s with 100 µ M EGTA to suppress Ca^2+^ entry. Under these conditions we detected a significant decrease in Ca^2+^ rise by either xestospongin C or ruthenium red treatment (Fig 6C). Downregulation of *TcPiezo2* expression by tetracycline addition (+Tet) had more dramatic effects (Fig 6D). To test *TcPiezo-CKO* responses to hyperosmotic stress, *TcPiezo Tet-OFF* epimastigotes expressing jGCaMP7s were submitted to hyperosmotic condition (800 mOsm) by adding D-mannitol (1,300 mOsm), but no intracellular Ca^2+^ increase was detected (S10 Fig). In summary, both TcPiezo1 and TcPiezo2, as well as the IP_3_R, contribute to the increase in intracellular Ca^2*+*^ levels of epimastigotes when they are submitted to hypoosmotic stress.

## Discussion

**Fig 7:**
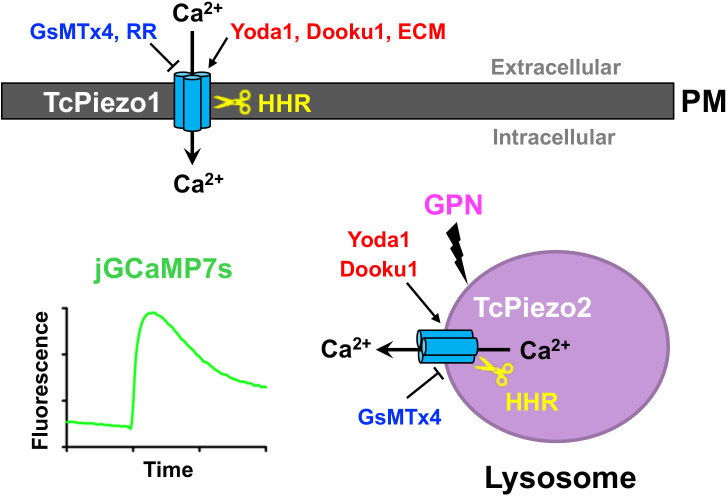
Scheme of TcPiezo-mediated Ca^2^^+^ entry and intracellular Ca^2^^+^ release in *T. cruzi.* TcPiezo 1 mediates Ca^2+^ entry through the plasma membrane (PM), which is activated by Yoda1, Dooku1 and extracellular matrix (ECM), and inhibited by spider mechanotoxin 4 (GsMTx4) and ruthenium red (RR), respectively. TcPiezo 2 mediates Ca^2+^ release from the lysosomes, which is activated by Yoda1and Dooku1, and blocked by GsMTx4, respectively. Intracellular Ca^2+^ changes are detected by using the cytosolic expressing jGCaMP7s. Downregulation of *TcPiezo1* or *TcPiezo2* by a novel conditional expression system using hammerhead ribozymes (HHR)-Shark1 and Shark3 establishes the essentiality of Piezo channels for the life cycle of *T. cruzi*.

We report here that *T. cruzi* possesses two mechanosensitive Piezo channels, named TcPiezo1 and TcPiezo2, with different subcellular localizations but similarly essential for normal proliferation, differentiation, and infectivity. While TcPiezo1 localizes to the plasma membrane and is involved in Ca^2+^ influx, TcPiezo2 localizes to the reservosomes and lysosomes and is important for Ca^2+^ release from these acidic compartments, which are therefore identified as acidic Ca^2+^ stores in trypanosomes. *T. cruzi* Piezo channels are activated by the small molecules Yoda1 and Dooku1, unaffected by Jedi1/Jedi2, inhibited by GsMTx4, and necessary for the intracellular Ca^2+^ increase that occurs upon mechanical stimulation by ECM, or by hypoosmotic stress. The essentiality of TcPiezo channels for the normal life cycle and Ca^2+^ homeostasis of *T. cruzi* is validated by the conditional downregulation of each of these genes using tetracycline and theophylline induction of aptazymes Shark1 and Shark3. Fig 7 shows a summary of the findings.

*T. cruzi* is a flagellated unicellular microorganism that is under continuous movement and therefore under mechanical shear stress. These characteristics explain the role of TcPiezo1 in Ca^2+^ entry under physiological conditions. Trypanosomes are known to possess a putative voltage gated Ca^2+^ channel (Ca_v_, VGCC) in the plasma membrane [43,53] and experiments in which the Ca_v_ inhibitor nifedipine was used indicate that both TcPiezo1 and TcCa_v_ are important for Ca^2+^ entry. Nifedipine had an additive effect on Ca^2+^ entry inhibition when *TcPiezo1* was downregulated. In addition, downregulation of *TcPiezo1* expression reduced Ca^2+^ entry by contact with ECM, a known mechano-stimulant of Piezo channels [5], indirectly supporting the mechanosensitive nature of this channel.

The presence of Piezo channels in endolysosomes has not been reported before although they are present in the plant vacuole [14], which have similarities with lysosomes. Lysosomes are considered important acidic Ca^2+^ stores [54] possessing, in case of animal cells, a luminal calcium concentration (500 µ M) [55,56] equivalent to that present in the endoplasmic reticulum, and Ca^2+^ channels like the two-pore channel (TPC) [57–59] and the transient receptor potential mucolipin (TRPML) channels [60]. Interestingly, two TRP channels have been localized to reservosomes and lysosomes of *T. cruzi* [61], one of them, TcTRP1 is an ortholog to the *T. brucei* mucolipin channel [62], while the other, TcTRP2, is annotated as putative polycystin cation channel. The endolysosomal localization of TcPiezo2 provides further evidence of their relevance as Ca^2+^ stores. Reservosomes are acidic pre-lysosomal compartments first described in the epimastigote stage of *T. cruzi* [63]. They are usually located in the posterior region of these cells and are rich in peptidases, like cruzipain [45] and serine carboxypeptidase [46], and lipids [64]. They also accumulate macromolecules taken up by endocytosis, such as transferrin, albumin, and low-density lipoprotein [63,65–67]. When epimastigotes transform into metacyclic trypomastigotes reservosome numbers decrease and they were proposed to have a role in the storage of nutrients needed for this differentiation [63].

*T. cruzi* transits between intracellular and extracellular forms, which are subjected to different stresses. During hypoosmotic stress, both TcPiezo1 and TcPiezo2 contribute to intracellular Ca^2+^ rise in epimastigates, suggesting that membrane stretch from osmotic swelling can directly activate TcPiezo-dependent Ca^2+^ flux. mPiezo1 activation upon hypo-osmotically induced cell swelling has been found in a variety of cells such as rat beta cells and HEK293 cells [68]. Recently, the conformational changes of the blades associated with mPiezo1 activation have been directly observed through the induction of cell swelling [69]. In contrast, during hyperosmotic stress, no intracellular Ca^2+^ increase in epimastigotes is detected, indicating that membrane curvature from osmotic shrinkage cannot activate the TcPiezo or other channels.

Conditional downregulation of *TcPiezo1* or *TcPiezo2* channel expression significantly affected epimastigotes proliferation and differentiation, trypomastigotes invasion of host cells, and intracellular proliferation of amastigotes. These results suggest the potential of these channels as drug targets.

Several nonspecific blockers of Piezo channels, like ruthenium red (RR) and GsMTx4 were used to test the sensitivity of TcPiezo channels. RR is known to block many TRP channels [70] and is not cell permeable. The lack of TRP channels in the plasma membrane of *T. cruzi* and its effect when applied from the extracellular side suggest that it might be specifically inhibiting TcPiezo1 channel activity. GsMTx4 is a peptide originally isolated from the venom of a tarantula and is the only inhibitor that specifically targets cation mechanosensitive channels [71]. It may act indirectly via the lipid bilayer [50,71,72]. It is also proposed to inhibit mPiezo1 channel activity by compacting the blades to release membrane-bending stress [69]. GsMTx4 was able to inhibit both TcPiezo1 and TcPiezo2 activity. Yoda1 was the first discovered mammalian Piezo1 (mPiezo) activator that induces channel opening in the absence of mechanical force by expanding the blades to make conformational movement upon binding but it does not activate mPiezo2 [49,69]. Dooku1 is an analogue of Yoda1 that lacked agonist activity on mPiezo1 and antagonize Yoda1 [50]. Interestingly, in contrast with the two mammalian Piezo channels, the small molecules Yoda1 and Dooku1 activated both TcPiezo channels, as detected by expressing jGCaMP7s in the transgenic parasites. This activation was not detected upon downregulation of the channels. Finally, the compounds Jedi1 and Jedi2 are mPiezo1 but not mPiezo2 activators discovered more recently [52]. Both Jedi1 and 2 are not cell permeable and activate mPiezo1 only from the extracellular side. They do not have structural similarity to Yoda1 implying distinct activation mechanisms and were inactive on TcPiezo channels.

As it has been reported before [47], addition of GPN resulted in Ca^2+^ release from lysosomes. The lower Ca^2+^ release when *TcPiezo2* expression was downregulated (Fig 4A) indicates that less Ca^2+^ was available in the lysosomes of these cells, which is consistent with the lysosomal localization of TcPiezo2 and suggest that this channel is required for GPN-induced Ca^2+^ release. However, no changes in Ca^2+^ release were observed when an inhibitor of the SERCA-ATPase (cyclopiazonic acid, Fig 4B) was added or Ca^2+^ entry was allowed (Fig 4C), again in agreement with the lysosomal localization of the channel. Interestingly, downregulation of *TcPiezo2* expression also resulted in less Ca^2+^ release under hypoosmotic conditions (Fig 6D).

In summary, our novel approach of using GECIs in transgenic parasites in which downregulation of gene expression can be induced allowed us to identify the essential role of these Piezo channels in Ca^2+^ entry and Ca^2+^ release from intracellular stores under physiological conditions and under mechanical and hypoosmotic stress.

## Methods

### Cell culture

*T. cruzi* epimastigotes (Y strain) were grown at 28°C in liver infusion tryptose (LIT) medium (0.5% [w/v] liver infusion, 0.5% [w/v] tryptose, 68 mM NaCl, 56 mM Na_2_HPO_4_, 5.4 mM KCl, 0.2% [w/v] glucose, 0.002% [w/v] hemin, pH 7.3 [73], supplemented with 10% (v/v) heat-inactivated newborn calf serum (NCS-HI) and 1% Gibco’s penicillin/streptomycin (10 kU/mL). Transgenic *T. cruzi* cell lines were maintained in LIT medium with appropriate antibiotics (250 μg/ml G418, 10 μg/ml blasticidin, 5μg/ml puromycin). Conditional knockout (CKO) *T. cruzi* was induced with 5 μg/mL fresh tetracycline or 250 μg/mL theophylline when the cells were at a density of 2 × 10^6^ epimastigotes/mL. The growth rate of epimastigotes was determined by counting cells in a Neubauer chamber.

Tissue culture cell-derived trypomastigotes and amastigotes were obtained as previously described [3]. Vero cells were grown in RPMI 1640 medium supplemented with 10% fresh fetal bovine serum (fresh FBS) and 1% Gibco’s penicillin/streptomycin (10 kU/mL) and incubated at 37°C with 5% CO_2_.

### *In vitro* metacyclogenesis

Metacyclic trypomastigotes were obtained as described [74] with minor modifications. Mid-log epimastigotes were collected by centrifugation at 1,000 × g for 7 min and washed twice in triatomine artificial urine (TAU) medium (190 mM NaCl, 17 mM KCl, 2 mM MgCl_2_, 2 mM CaCl_2_, 8 mM sodium phosphate buffer pH6.0), and incubated in TAU for 2 hours at 28^o^C. The parasites were then diluted 100-fold in TAU 3AAG medium (TAU supplemented with 20 mM L-proline, 10 mM glucose, 50 mM sodium glutamate, and 2 mM sodium aspartate) in T25 flasks and incubated horizontally with or without 5 μg/mL fresh tetracycline or 250 μg/mL theophylline at 28^o^C for 96 hours.

For quantification, assays samples were fixed for 1 hour at RT in 4% paraformaldehyde in PBS, attached to poly-L-lysine-coated coverslips, and washed three times with PBS. Then, parasites were incubated for 1 hour in 50 mM NH_4_Cl in PBS, washed three times in PBS, and mounted onto glass slides with Fluoromount-G containing 15 μg/mL 4’,6-diamidino-2-phenylindole (DAPI), which stains the DNA present in the nucleus and the kinetoplast of parasites. Twenty fields/slides were analyzed on an Olympus IX-71 inverted fluorescence microscope with a 100 × objective in three independent experiments. Metacyclic trypomastigotes were distinguished from epimastigotes by the location of the kinetoplast in the cell body (posterior in metacyclic trypomastigotes; between the nucleus and the flagellum in epimastigotes). Metacyclic trypomastigotes without tetracycline or theophylline treatment were also collected to initially infect Vero cells to generate tissue culture-derived trypomastigotes.

### *In vitro* infection assays

*T. cruzi* invasion and intracellular replication assays were performed as previously described [24], with some modifications. Gamma-irradiated (2,000 rads) Vero cells (4.5 x 10^5^ cells) were plated onto sterile coverslips in a 12-well plate and incubated overnight at 37°C in 5% CO_2_ in RPMI medium plus 10% fresh FBS. Tissue culture-derived trypomastigote collections were incubated with or without fresh tetracycline (5 µg/ml) or theophylline (250 µg/ml) at 4°C overnight and then 1 hour at 37^o^C to allow amastigotes to settle from swimming trypomastigotes. Trypomastigotes from the supernatants of these collections were counted and used to infect the mammalian cells at a 10:1 ratio of trypomastigotes to host cells in the absence and presence of tetracycline or theophylline. At 4 h post-infection, coverslips were washed extensively with Hanks’ solution, followed by PBS at pH 7.4 to remove any extracellular parasites. Coverslips were fixed immediately in 4% paraformaldehyde in PBS (pH 7.4) at 4°C for 30 min, washed once with PBS, and mounted onto glass slides in Fluoromount-G containing 15 µg/ml of DAPI, which stains host and parasite DNA (S4F, S4G, S7G, and S7H Figs). Coverslips were analyzed on an Olympus IX-71 inverted fluorescence microscope to quantify the number of host cells that contained intracellular parasites and the number of intracellular parasites per cell in 40 randomly selected fields. Three hundred host cells were counted per sample in three independent experiments.

To quantify amastigote replication, Vero cells were infected at a 10:1 ratio of tissue culture-derived trypomastigotes to host cells, and after they were washed at 4 h post-infection as described above, coverslips were allowed to incubate in the absence and presence of tetracycline (1 µg/ml) or theophylline (125 µg/ml) for 72 h post-infection at 37°C with 5% CO_2_ prior to fixation and DAPI staining. Coverslips were mounted onto glass slides and analyzed on an Olympus IX-71 inverted fluorescence microscope. Amastigotes in infected host cells were counted using a 100 × objective.

To confirm that expression down-regulation of *TcPiezos* could be achieved in amastigotes and trypomastigotes we added 1 µg/ml tetracycline or 125 µg/ml theophylline to cultures releasing large amounts of these forms. After 2 days the cells were collected, trypomastigotes were separated from amastigotes by decantation (1 hour at 37^o^C incubation as above) and analyzed by western blot (S11 Fig).

### Cell transfections

*T. cruzi* transfections were performed as previously described [25]. Mid-log phase *T*. *cruzi* Y strain epimastigotes (5 x 10^7^ cells) were washed with sterile PBS, pH 7.4, at room temperature (RT) and transfected in ice-cold Cytomix buffer (120 mM KCl, 0.15 mM CaCl_2_, 10 mM K_2_HPO_4_, 25 mM Hepes, 2 mM EDTA, 5 mM MgCl_2_, pH 7.6) containing 10–25 μg of each DNA construct in 4-mm electroporation cuvettes with three pulses (1500 volts, 25 μF each pulse) delivered by a Gene Pulser Xcell Electroporation System (Bio-Rad). Stable cell lines were obtained and maintained under drug selection with appropriate antibiotic(s) (250 μg/ml G418, 10 μg/ml blasticidin, 5 μg/ml puromycin). Transfected epimastigotes were cultured in LIT media supplemented with 20% NCS-HI until stable and clonal cell lines were established.

### Knockout of *TcPiezo1* and *TcPiezo2*

Single guide RNA (sgRNA) sequences to target the pore region or the whole *TcPiezo1* and *TcPiezo2* (TryTripDB gene ID numbers TcYC6_0088320, and TcYC6_0007880, respectively), as described in S3A Fig, were PCR amplified from plasmid pUC_sgRNA [38]. Selection of the protospacers was performed using EuPaGDT (Eukaryotic Pathogen CRISPR guide RNA Design Tool [http://grna.ctegd.uga.edu/]). The protospacer sequences were included into the forward primers (TcPZ1gRNA-koF1/-koF2, and TcPZ1gRNA-koF1/-koF2/-koF3), while a common reverse primer (Com-gRNA-R) (S1 Table) was used for sgRNA amplification. These primers also contained a BamHI restriction site for cloning into Cas9/pTREX-n [38] to generate the *TcPiezo* sgRNA/Cas9/pTREX-n constructs. The sgRNA orientation was verified by PCR using the *TcPiezo*-sgRNA forward primers and the HX1 reverse primer (HX1-R) (S1 Table). Positive clones that generate a 190-bp PCR fragment were also sequenced. DNA donor cassettes designed to promote homologous directed repair and replacement of the pore region or the whole *TcPiezo* genes were obtained by PCR using a set of long primers, containing 120 bp homology arms which correspond to the *TcPiezo* genes, as indicated in S3A Fig, with 20–22 nt annealing on blasticidin (*Bsd*) or puromycin (*Puro*) resistance gene (S1 Table). The *TcPiezo-*sgRNA/Cas9/pTREX-n constructs and their corresponding DNA donor cassettes were co-transfected into *T. cruzi* epimastigotes and then selected with 250 μg/ml G418 and 10 μg/ml blasticidin or 5 μg/ml puromycin for 5 weeks to obtain antibiotics resistance cell lines.

### Conditional knockout of *TcPiezo1* and *TcPiezo2*

Downregulation of *TcPiezo1* or *TcPiezo2* was developed by a novel conditional expression system for *T. cruzi* using modified hammerhead ribozymes (HHR) (S3F Fig), named as Small Hammerhead Aptazyme-Regulated Knockdown-SHARK^31^. First, *TcPiezo1* and *TcPiezo2* aptazyme tagging cassettes (composed of 3 × Ty tag, *Tet-OFF/Theo-OFF* aptazyme sequence, GAPDH 3’UTR, and *Bsd* gene) were generated by PCR using the pMiniTrex-mCherry-aptazymes (Shark1 and Shark3) [31] as templates with gene-specific forward and reverse primers TcPZ1-kd-F and TcPZ1-kd-R, TcPZ2-kd-F and TcPZ2-kd-R (S1 Table), respectively. The forward and reverse primers contain 50 bp homology arms corresponding to regions close to the 3’ ends of ORFs (right upstream of the Cas9 sgRNA and downstream of the stop codon of *TcPiezo1*, and upstream of the stop codon and downstream of the Cas9 sgRNA of *TcPiezo2*) (S3B Fig), followed in frame by the 23–24 nucleotides (nt) of the backbone sequences of pMiniTrex-mCherry-aptazymes (Shark1 and Shark3). Second, chimeric sgRNA targeting 25 nt upstream of the stop codon of *TcPiezo1* and 24 nt downstream of the stop codon of *TcPiezo2* (S3B Fig) were PCR-amplified and cloned into Cas9/pTREX-n vector to generate the gene specific sgRNA/Cas9/pTREX-n vectors, as described above. Last, gel-purified *TcPiezo1* and *TcPiezo2* aptazyme cassettes (15 μg) and the corresponding sgRNA/Cas9/pTREX-n (15 μg) were co-transfected into *T. cruzi* epimastigotes, respectively, to generate *TcPiezo Tet-OFF/Theo-OFF* (also called as *TcPiezo-CKO,* or *TcPiezo-Shark1/3*) homozygous transgenic cell lines, which were confirmed by PCR (S3C–E Fig) using the pair primers TcPZ1-ORF-F and TcPZ1–3UTR-R, and TcPZ2-ORF-F and TcPZ2–3UTR-R (S1 Table), respectively.

### PCR-mediated *in situ* epitope-tagging of TcPiezo2

The one-step epitope-tagging protocol reported by Oberholzer *et al*. [75] was used to produce C-terminal smV5 tagging cassette of TcPiezo2 for transfection of *T. cruzi* epimastigotes. In brief, the PCR forward and reverse primers (S1 Table) included terminal 142 nt of ORF before its stop codon and the reverse complement of the 112 nt of the 3’UTR respectively, followed in frame by the 21–26 nucleotides of the backbone sequences of pMOTag2mV vector [44]. The smV5 tagging cassette containing an antibiotic selection marker (puromycin resistance gene) was generated for cell transfection by PCR using the pMOTag2mV as template with the primers TcPiezo2-TF and TcPiezo2-TR (S1 Table).

### Expression of GCaMP Ca^2+^ sensors in *T. cruzi*

The coding sequences of genetically encoded Ca^2+^ indicators (GECIs) GCaMP6f, GCaMP6s, jGCaMP7f and jGCaMP6s (composed of 6 × His-T7 epitope-Xpress tag, circularly permuted GFP, the Ca^2+^-binding protein calmodulin (CaM), and CaM-interacting M13 peptide) were PCR-amplified by using pGP-CMV-GCaMP6f/s [41] or pGP-CMV-jGCaMP7f/s [42] as template, respectively, with primers GCaMP-F and GCaMP-R (S1 Table). The PCR products were gel-purified, XbaI-HindIII-digested, and then clone into *Xba*I-HindIII-cut pTREX-n or pTREX-p [25], which contains a neomycin (G418) or puromycin resistance gene, respectively. After confirmed by sequencing, the correct GCaMP variant constructs were transfected into *T. cruzi* WT or *TcPiezo-CKO* cell lines. The transgenic cell lines constitutively expressing cytosolic GCaMP Ca^2+^ sensors were confirmed by immunoblotting and IFA assays and used for Ca^2+^ entry and release assays, as described below.

### Ca^2+^ entry and release assays

(i) **Fura-2/AM**

Tissue culture cell-derived *TcPiezo1-CKO* amastigotes in the absence or presence of tetracycline or theophylline for 2 days were collected in 50 ml sterile centrifuge tube and settled by incubation at 4^o^C overnight to separate from swimming trypomastigotes. Amastigotes were washed twice with buffer A with glucose (BAG), which contained 116 mM NaCl, 5.4 mM KCl, 0.8 mM MgSO_4_, 5.5 mM D-glucose and 50 mM Hepes at pH7.3, at 1000 × g for 7 min at room temperature and loaded in BAG with 5 μM Fura-2/AM (Molecular Probes) plus 1.5% sucrose for 30 min at a 30°C water bath with mild agitation and rinsed twice by centrifugation to remove extracellular dye. The cells were resuspended to a final density of 1 x 10^9^ cells per ml in BAG. For fluorescence measurements, a 50 μl-aliquot (5 × 10^7^ cells) of the cell suspension was added to 2.45-ml BAG containing 100 μM EGTA (<10 μM extracellular Ca^2+^) in a cuvette. Ca^2+^ entry was monitored by adding 1.8 mM Ca^2+^ to suspension of amastigotes in the cuvette on a Hitachi F7000 spectrofluorometer with excitation wavelength 340/380 nm and emission wavelength 510 nm on the Hitachi F7000 spectrofluorometer, as previously described [76].

(ii) **GCaMPs**

*TcPiezo-CKO* epimastigotes or trypomastigotes expressing GCaMP-type Ca^2+^ indicators were incubated in the absence or presence of tetracycline (or theophylline) for 2 days. The epimastigotes were collected by centrifugation at 1000 × g for 7 min at RT. Tissue culture cell-derived trypomastigote collections were incubated at 4°C overnight to allow amastigotes to settle from swimming trypomastigotes. Trypomastigotes from the supernatants were collected by centrifugation at 1,600 × g for 7 min. The epimastigotes and trypomastigotes were rinsed twice, resuspended to a final density of 1 x 10^9^ cells per ml in BAG, and kept on ice. For intracellular Ca^2+^ measurements, a 50 μl-aliquot (5 x 10^7^ epimastigotes or 2 × 10^7^ trypomastigotes) of the cell suspensions was added to 1.95-ml BAG in a cuvette on a Hitachi F7000 spectrofluorometer with excitation wavelength 488 nm and emission wavelength 510 nm on the Hitachi F7000 spectrofluorometer. Ca^2+^ entry was monitored in the *TcPiezo1-CKO* cells by adding 1.8 mM Ca^2+^, whereas Ca^2+^ release was detected in the *TcPiezo2-CKO* cells by adding 70 μM glycyl-l-phenylalanine 2-naphthylamide (GPN). Ca^2+^ ionophore (ionomycin), Piezo activators (Yoda1, Dooku1, Jedi1, Jedi2, Extracellular Matrix-ECM), Piezo inhibitor (*Grammostola spatulata* mechanotoxin 4 (GsMTx4), or SERCA inhibitor cyclopiazonic acid (CPA) were also added as described in Figures. Ca^2+^ fluxes were measured using jGCaMP7s signal fluorescence changes (*∆*) in arbitrary units of fluorescence (AU), which were obtained by subtracting the values at the turning point of the baseline from those at the peaks (S1 Data).

### Osmotic stress assays

*TcPiezo Tet-OFF* epimastigotes expressing jGCaMP7s were grown in the absence and presence of 5 μg/mL tetracycline for 2 days. Exponentially growing parasites were collected by centrifugation at 1,000 *g* for 7 min and washed twice with BAG. Cells were resuspended to a final density of 1 × 10^9^ cells per ml in the isosmotic BAG buffer (300 mOsm) and kept on ice. A 50-μl aliquot (5x10^7^ cells) of cell suspension was added to the 550-μl isosmotic buffer and incubated without or with 100 μM EGTA. Osmolarity of the reaction buffer was changed by adding 600 μl of Millipore H_2_O to achieve the hypoosmotic (150 mOsm) condition or by adding 600 μl of 1,300 mOsm D-mannitol (Sigma Aldrich) to make the hyperosmotic (800 mOsm) condition. The cells were incubated with 100 μM EGTA to remove extracellular Ca^2+^. 1 μM Xestospongin C (Xest-C) was added to monitor Ca^2+^ release from acidocalcisomes by blocking inositol 1,4,5-trisphosphate (IP_3_) receptor. 4 μM ruthenium red (RR) was added. The cytosolic Ca^2+^ concentration of cells was monitored under hypoosmotic or hyperosmotic stress in a fluorometer with excitation at 488 nm and emission at 510 nm, as described above.

### Immunofluorescence microscopy

*T. cruzi* cells were washed twice with PBS and fixed with 4% paraformaldehyde in PBS pH 7.4 for 1 hour at RT. The fixed cells were washed twice with PBS, allowed to adhere to poly-L-lysine-coated coverslips and permeabilized with 0.3% Triton X-100/PBS for 3 min for the parasites. After blocking with PBS containing 3% BSA, 1% fish gelatin, 50 mM NH_4_Cl and 5% goat serum for 1 h, cells were stained in 1% BSA/PBS with the purified clone BB2 monoclonal antibody against Ty1 (1:100), monoclonal antibody against V5 (1:100), monoclonal antibody against His (1:100), rabbit polyclonal antibody against *T. cruzi* H^+^ -ATPase (α-TcHAf, 1:200), rabbit polyclonal antibody against *T. cruzi* cruzipain (α-TcCZP, 1:2,500), rabbit polyclonal antibody against *T. cruzi* serine carboxypeptidase (α-TcSCAR, 1:1,000), or rabbit polyclonal antibody against GFP (1:100) for 1 h. After thoroughly washing with PBS containing 3% BSA, cells were incubated with Alexa 488-conjugated goat anti-mouse or anti-rabbit, and Alexa 546-conjugated goat anti-rabbit antibody at 1:1,000 for 1 h. The cells were counterstained with DAPI before mounting with Gold ProLong Gold antifade reagent (Molecular Probes). Differential interference contrast and fluorescent optical images were captured using an Olympus IX-71 inverted fluorescence microscope with a Photometrix CoolSnap^HQ^ charge-coupled device camera driven by DeltaVision software (Applied Precision, Seattle, WA). Images were deconvolved for 15 cycles using Softwarx deconvolution software. Pearson’s correlation coefficients were calculated using the Softwarx software by measuring the whole-cell images.

### Western blot analyses

*T. cruzi* cells were harvested and washed twice in BAG. The washed cells were lysed with RIPA buffer (150 mM NaCl, 20 mM Tris/HCl, pH 7.5, 1 mM EDTA, 1% SDS, and 0.1% Triton X-100) containing protease inhibitor tablet in ice for 1 h. The protein concentration was determined by using Pierce BCA protein assay kit with the microplate reader. Total cell lysates were mixed with 2 × Laemmli sample buffer (BioRad) at 1:1 ratio (volume/volume) and directly loaded. The separated proteins were transferred onto nitrocellulose membranes using a Bio-Rad transblot apparatus. The membranes were blocked with 10% non-fat milk in PBS-T at 4^o^C overnight. The blots were incubated with mouse antibodies against Ty1 (1:2,500), rabbit antibodies against GFP (1:2,500), mouse antibodies against V5 (1:2,500), monoclonal antibody against His (1:1,000), and mouse antibodies against tubulin (1:10,000) for 1 h. After five washings with PBS-T, the blots were incubated with horseradish peroxidase conjugated anti-mouse or anti-rabbit IgG (H + L) antibody at a dilution of 1:15,000 for 1 h. After washing five times with PBS-T, the immunoblots were visualized using Pierce ECL Western blotting substrate according to the manufacturer’s instructions. Full-size images of immunoblots are shown in S12 Fig.

## Supporting information

S1 Fig Multiple sequence alignment. The C-terminal conserved regions of TcPiezo1 and TcPiezo2 (TriTrypDB: TcYC6_0088320/KAF8295942 and TcYC6_0007880/KAF8281887, respectively) and mPiezo1 and mPiezo2 (GenBank accession no. NP_001344278.1 and NM_001039485, respectively) [33] were aligned with MUSCLE (https://www.ebi.ac.uk/Tools/msa/muscle) and the webserver site (http://www.bioinformatics.org/sms/index.html) [77]. Identical (*black*) and similar (*gray*) amino acid residues are shaded. The groups of similar amino acids (ILV, FWY, KRH, DE, GAS, P, C, TNQM) were used for the similarity calculation. The topology from TM34 to TM38 was derived from the structure of mPiezo1 [78]. The PFEW motif boxed in pink is conserved among plants, mammals and protozoa [7]. In mPiezo1 and mPiezo2, TM37 and TM38 are defined as outer helix (OH) and inner helix (IH) of the pore region, respectively. The C-terminal extracellular domain (CED) consists of 4 alpha domains (α1-α4) and 9 beta domains (β1-β9). The conserved residues boxed in red and green in the IH (TM38)-C-terminal domain (CTD) region form the transmembrane (TM) gate and cytosolic constriction neck of the Ca^2+^-transducing pore [34–36].
(PDF)

S2 Fig AlphaFold II structure predictions. The structures of C-terminal conserved sequences (S1 Fig) of mPiezo1 (A), mPiezo2 (C), TcPiezo1 (B), and TcPiezo2 (D), were predicted with AlphaFold II [37]. A ribbon diagram of the pore region, formed by OH (TM37) and IH (TM38) of Piezo. The specific regions or domains are labelled: alpha domains (α1-α4); beta domains (β1-β12); OH, outer helix; anchor; IH, inner helix; CTD, C-terminal domain; IH-CTD linker; TM34–36.
(PDF)

S3 Fig Knockout (KO) and conditional KO of *TcPiezo1* or *TcPiezo2.* (A) Schematic representation of the strategy used to generate TcPiezo-KO mutants by CRISPR/Cas9-induced homologous recombination. Single guide (sg) RNAs (sgRNA1, sgRNA2, sgRNA3) were generated with Cas9 endonuclease to bind and cut the Piezo pore region (boxed) or the whole *TcPiezo1* or *TcPiezo2* genes. DNA was repaired or replaced with a blasticidin (*bsd*) or puromycin (*puro*) resistance gene cassette, which was generated by PCR using a set of forward and reverse primers (TcPZ-bsd-koF and TcPZ-bsd-koR, TcPZ-puro-koF and TcPZ-puro-koR, S1 Table) containing 120-bp homologous regions from the *TcPiezo* loci. (B) Schematic diagram of CRISPR/Cas9 mediated endogenous C-terminal tagging of *TcPiezo* with an aptazyme cassette (composed of 3 × Ty tag, ribozyme, GAPDH 3’UTR, and *Bsd* gene), which was generated by PCR with primers TcPZ-kd-F and TcPZ-kd-R (S1 Table). (C) The aptazyme cassette integrated into the C-terminal loci of *TcPiezo* by homologous recombination. *TcPiezo* tagging was verified by PCR using primers TcPZ-ORF-F and TcPZ-3UTR-R (S1 Table). The intact loci generated a PCR product of ~ 0.8 kb from parental Y strain while the tagged loci generated a fragment of ~ 1.9 kb from a homozygous cell line. (D) PCR analysis showing that both loci of *TcPiezo1* were tagged with aptazyme *Tet-OFF* or *Theo-OFF*. (E) PCR analysis showing that both loci of *TcPiezo2* were tagged with aptazyme *Tet-OFF* or *Theo-OFF*. (F) Illustration of the effect of tetracycline (Tet) or theophylline (Theo) binding to hammerhead ribozyme (HHR) on *TcPiezo* mRNA stability.
(PDF)

S4 Fig Characterization of *TcPiezo1-CKO.* (A) Western blot analyses of *TcPiezo1 Tet-OFF* and *TcPiezo1 Theo-OFF* epimastigotes grown in the absence of tetracycline (-Tet) or theophylline (-Theo). Total lysates (30 μg) were subjected to 10% SDS-polyacrylamide gel electrophoresis before transfer to a nitrocellulose membrane and then stained with antibodies against Ty1 (top). One band of ~ 290 kDa was detected in epimastigote homogenates. Membranes were stripped and re-incubated with antibody against Alpha-tubulin as a loading control (bottom, Tub). (B) Growth of *TcPiezo1 Theo-OFF* epimastigotes in the absence (black line, *-Theo*) or presence (red line, *+ Theo*) of 250 μg ml^−1^ theophylline for the indicated number of days. Western blot analyses of *TcPiezo1 Theo-OFF* epimastigotes grown in the absence (0) or presence (2–6) of theophylline. Total lysates were subjected to 10% SDS-PAGE before transfer to a nitrocellulose membrane and stained with Ab against Ty1. Bands of 290 kDa were detected. Alpha-tubulin was used as loading control. (C) Percentage of metacyclic trypomastigotes in *TcPiezo1 Theo-OFF* epimastigote cultures after incubation in TAU 3AAG medium in the absence (-Theo) and presence (+Theo) of 250 µ g/ml theophylline after 96 h. (D) Effect of non-induced (-Theo) and induced (+Theo) *TcPiezo1 Theo-OFF* on trypomastigote infection of Vero cells after 4 h. (E) Effect of non-induced (-Theo) and induced (+Theo) *TcPiezo1 Theo-OFF* on amastigote replication after 72 h. In panels B, C, D, E, values are mean ± s.d. (*n* = 3). One-way ANOVA with multiple comparisons (*P < 0.05, **P < 0.01, ***P < 0.001). (F) Representative images of Vero cell infected with Tet-induced (+Tet) and non-induced (-Tet) *TcPiezo1 Tet-OFF* trypomastigotes. (G) Representative images of Vero cell infected with Theo-induced (+Theo) and non-induced (-Theo) *TcPiezo1 Theo-OFF* trypomastigotes. In panels F and G, nuclei and kinetoplasts were DAPI stained. Scale bars 10 µm.
(PDF)

S5 Fig Downregulation of *TcPiezo1* expression reduces Ca^2+^ entry of *T. cruzi.* (A, B) Ca^2+^ entry was affected by *TcPiezo1* downregulation in *T. cruzi* amastigotes. Fura-2/AM loaded induced (+Tet/ + Theo) or uninduced (-Tet/-Theo) amastigotes were suspended in buffer with 100 µ M EGTA and 1.8 mM CaCl_2_ were added at 150 sec (arrows). Cytosolic Ca^2+^ concentrations in the tissue-derived amastigotes were quantified in nM. (C, D) Intracellular Ca^2+^ by *TcPiezo1 Theo-OFF* epimastigotes (C) or trypomastigotes (D) expressing jGCaMP7s in AU. (C) Addition of 1.8 mM Ca^2+^ in Theo-induced *TcPiezo1* cells (+Theo) elicited a lower increase in intracellular Ca^2+^ than in non-induced cells (-Theo). (D) ECM triggered TcPiezo1-mediated Ca^2+^ entry in trypomastigotes. Addition of 40 µg ECM to non-induced (-Theo) trypomastigotes significantly increased intracellular Ca^2+^, compared to Theo-induced cells (+Theo). In panels C-D, addition of BAG (baseline, B), instead of 1.8 mM Ca^2+^, was used as a control. Values are means ± s.d. (n = 3). One-way ANOVA with multiple comparisons (* *P* < 0.05, **P < 0.01, ***P < 0.001).
(PDF)

S6 Fig Expression of GCaMP Ca^2+^ sensors in *T. cruzi.* (A-C) Expression of GCaMP Ca^2+^ sensors in *T. cruzi* WT. (A) Western blot analysis of lysates labels a band of 50 kDa with antibody against GFP. Alpha-tubulin was used as loading control. (B) Representative traces of epimastigotes expressing GCaMP6f (6f), GCaMP6s (6s), jGCaMP7f (7f), and jGCaMP7s (7s) incubated in BAG, showing extracellular and intracellular Ca^2+^ responses to 1 µ M ionomycin. (C) Fixed epimastigotes expressing GCaMP Ca^2+^ sensors (GCaMP6f, GCaMP6s, jGCaMP7f, and jGCaMP7s) assayed by immunofluorescence microscopy with antibody against GFP showing cytosolic localization. Scale bars 10 µm. (D-I) Expression of jGCaMP7s in *TcPiezo-CKO* epimastigotes (*TcPiezo1 Tet-OFF/Theo-OFF* (D-F) and *TcPiezo2 Tet-OFF/Theo-OFF* (G-I). (D, G) *TcPiezo Tet-OFF/Theo-OFF* epimastigotes expressing jGCaMP7s assayed by immunofluorescence microscopy with antibody against His showing cytosolic localization. DIC, differential interference contrast microscopy. Scale bars 10 µm. (E, H) Western blot analysis of *TcPiezo-CKO* lysates labels a band of 50 kDa with antibody against His. Alpha-tubulin was used as loading control. (F, I) Representative traces of *TcPiezo-CKO* epimastigotes expressing jGCaMP7s incubated in BAG, showing intracellular Ca^2+^ responses to 1 µ M ionomycin. 100 µ M EGTA was added to remove extracellular Ca^2+^.
(PDF)

S7 Fig Subcellular localization and characterization of *TcPiezo2 Theo-OFF.* (A) smV5-tagged TcPiezo2 colocalized with *T. cruzi* serine carboxypeptidase (TcSC) to the reservosomes/lysosomes of epimastigotes (PCC: 0.7024), trypomastigotes (PCC: 0.8293) and amastigotes (PCC: 0.7933). Yellow in merged images indicates colocalization. DIC, differential interference contrast microscopy. Scale bars 5 or 10 µm, as indicated. (B) Western blot analysis of C-terminally smV5-tagged TcPiezo2 epimastigotes. Total lysates (30 μg) were subjected to 10% SDS-polyacrylamide gel electrophoresis before transfer to a nitrocellulose membrane and then stained with antibodies against V5 (top). One band of ~ 320 kDa was detected in epimastigote homogenates. Membranes were stripped and re-incubated with antibody against Alpha-tubulin as a loading control (bottom). (C) Growth of *TcPiezo2 Theo-OFF* epimastigotes in the absence (black line, *− Theo*) or presence (red line, *+ Theo*) of 250 μg ml^−1^ theophylline for the indicated number of days. Western blot analyses of *TcPiezo2 Theo-OFF* epimastigotes grown in the absence (0) or presence (2–6) of theophylline. Total lysates were subjected to 10% SDS-PAGE before transfer to a nitrocellulose membrane and stained with Ab against Ty1. Bands of 280 kDa were detected. Alpha-tubulin was used as loading control. (D) Percentage of metacyclic trypomastigotes in *TcPiezo2 Theo-OFF* epimastigote cultures after incubation in TAU 3AAG medium in the absence (-*Theo*) and presence (+*Theo*) of 250 µ g/ml theophylline after 96 h. (E) Effect of non-induced (-*Theo*) and induced (+*Theo*) *TcPiezo2 Theo-OFF* on trypomastigote infection of Vero cells after 4 h. (F) Effect of non-induced (-*Theo*) and induced (+*Theo*) *TcPiezo2-CKO* on amastigote replication after 72 h. In panels C, D, E, F, values are mean ± s.d. (*n* = 3). One-way ANOVA with multiple comparisons (**P < 0.01, ***P < 0.001). (G) Representative images of Vero cell infected with Tet-induced (+Tet) and non-induced (-Tet) *TcPiezo2 Tet-OFF* trypomastigotes. (H) Representative images of Vero cell infected with Theo-induced (+Theo) and non-induced (-Theo) *TcPiezo2 Theo-OFF* trypomastigotes. In panels G and H, nuclei and kinetoplasts were DAPI stained. Scale bars 10 µm. (I) Ty1-tagged TcPiezo2 Tet-OFF colocalization with *T. cruzi* cruzipain (TcCZP) to the reservosomes/lysosomes of epimastigotes (PCC: 0.7892), as detected by immunofluorescence analyses with antibodies against Ty1. Yellow in merged images indicates colocalization. DIC, differential interference contrast microscopy. Scale bars 10 µm.
(PDF)

S8 Fig Effects of *TcPiezo2* expression downregulation on intracellular Ca^2+^ stores in *T. cruzi.* Intracellular Ca^2+^ concentrations of 5 × 10^7^ *TcPiezo2 Theo-OFF* epimastigotes expressing jGCaMP7s were measured by jGCaMP7s signal fluorescence in AU. (A, B) 100 µ M EGTA was incubated with *TcPiezo2 Theo-OFF* cells to remove extracellular Ca^2+^, abolishing TcPiezo1-mediated Ca^2+^ entry. (A) TcPiezo2 mediated lysosomal Ca^2+^ release. Addition of 70 µ M GPN for activation of lysosomal Ca^2+^ release in Theo-induced *TcPiezo2* cells (+Theo) elicited a lower increase in intracellular Ca^2+^ than in non-induced cells (-Theo).(B) Addition of 30 µ M CPA for activation of ER Ca^2+^ release demonstrated no significant difference between Theo-induced (+Theo) and non-induced (-Theo) cells. (C) Addition of 1.8 mM Ca^2+^ showed no significant difference in intracellular Ca^2+^ between Theo-induced (+Theo) and non-induced (-Theo) cells. (D) 100 µ M EGTA was added to remove extracellular Ca^2+^. Addition of 1 µ M ionomycin in Theo-induced cells (+Theo) elicited less increase of intracellular Ca^2+^ than in non-induced cells (-Theo). Addition of DMSO or BAG was used as control (baseline) labeled with B. Values are means ± s.d. (n = 3). One-way ANOVA with multiple comparisons (*P < 0.05).
(PDF)

S9 Fig Effects of Piezo activators and inhibitors on TcPiezo channels. Intracellular Ca^2+^ changes of 5 × 10^7^ *TcPiezo Theo-OFF* epimastigotes expressing jGCaMP7s were measured by jGCaMP7s signal fluorescence in AU. (A) Dooku1-evoked TcPiezo1-mediated Ca^2+^ entry. Two Ca^2+^ spikes were generated in Theo-induced (+Theo) and non-induced (-Theo) *TcPiezo1 Theo-OFF* cells, respectively, by the additions of 1.8 mM Ca^2+^ and 40 µ M Dooku1. Changes in jGCaMP7s fluorescence (first peak and second peak) in Theo induced (+Theo) and non-induced (-Theo) cells were shown in bar graphs. Downregulation of *TcPiezo1* expression significantly decreased Dooku1-evoked TcPiezo1-mediated Ca^2+^ entry (left bar graph) but had no effect on Dooku1-evoked TcPiezo2-mediated Ca^2+^ release (right bar graph). (B) 100 µ M EGTA was incubated with *TcPiezo2 Theo-OFF* cells to remove extracellular Ca^2*+*^, abolishing TcPiezo1-mediated Ca^2+^ entry. 70µ M GPN was added to activate Ca^2+^ release from lysosomes in the cells. Downregulation of *TcPiezo2* expression (+Theo) showed a significant decrease of lysosomal Ca^2+^ release as in S8A Fig. Addition of 10 µ M GsMTx4 showed a significant reduction of intracellular Ca^2+^ in non-induced cells (-Theo+GsM) like downregulation of *TcPiezo2* (+Theo) and had an additive effect on TcPiezo2-mediated Ca^2+^ release in Theo-induced cells (+Theo+GsM). Changes in jGCaMP7s fluorescence and the rates of fluorescence increase upon GsM inhibition in Theo-induced (+Theo±GsM) and non-induced (-Theo±GsM) cells were shown in left and right bar graphs, respectively. (C) 100 µ M EGTA and 70 µ M GPN were added to *TcPiezo2 Theo-OFF* cells (±Theo), as indicated. Addition of 20 µ M Yoda1 (+Yoda1) dramatically stimulated TcPiezo2-mediated Ca^2+^ release from lysosomes. Changes in jGCaMP7s fluorescence and the rates of fluorescence increase upon Yoda1 activation in Tet-induced (+Tet±Yoda1) and non-induced (-Te±Yoda1) cells were shown in left and right bar graphs, respectively. (D) Jedi 1 and Jedi2 did not activate TcPiezo1. Additions of 3 pulses of 200 µ M Jedi1 or Jedi2 at 100 s, 200 s and 300 s (arrows), respectively, to non-induced *TcPiezo1 Tet-OFF* epimastigotes (5 × 10^7^ cells) did not affect intracellular Ca^2+^ in the cells. Addition of DMSO, instead of Yoda1/Dooku1/GsM/Jedi1/Jedi2, was used as control (baseline) labeled with B. In bar graphs A, B, C, values are means ± s.d. (*n* = 3). One-way ANOVA with multiple comparisons (**P < 0.01, ***P < 0.001).
(PDF)

S10 Fig *TcPiezo-CKO* responses to hyperosmotic stress. *TcPiezo Tet-OFF* epimastigotes expressing jGCaMP7s (5 × 10^7^ cells) in 600 µl isosmotic buffer (300 mOsm) were submitted to hyperosmotic stress (800 mOsm) by adding 600 µl mannitol (1,300 mOsm) at 50 s. Under the hyperosmotic stress, the *TcPiezo1 Tet-OFF* (A) and *TcPiezo2 Tet-OFF* (B) cells shrank but no intracellular Ca^2+^ rose (red tracings). No intracellular Ca^2+^ was increased by adding 600 µl isosmotic BAG.
(PDF)

S11 Fig Western blot analysis of *TcPiezo Tet-OFF/Theo-OFF* trypomastigotes and amastigotes in the absence (-Tet/-Theo) or presence (+Tet/ + Theo) of tetracycline or theophylline for 2 days. Total lysates (30 μg) were subjected to 10% SDS-polyacrylamide gel electrophoresis before transfer to a nitrocellulose membrane and then stained with antibodies against Ty1 (top). One band of ~ 290 kDa (A) or ~ 280 kD (B) was detected in trypomastigotes (Trypo) and amastigotes (Ama) homogenates. Membranes were stripped and re-incubated with antibody against Alpha-tubulin as a loading control (bottom).
(PDF)

S12 Fig Full western blot analyses. (PDF)

S1 Table Primers used in this study. (PDF)

S1 Data Statistical treatment of the data. (XLSX)
