# Supplementary material for: Lysosome and plasma membrane Piezo channels of Trypanosoma cruzi are essential for proliferation, differentiation and infectivity
Source: PLoS Pathog. 2025 Apr 23;21(4):e1013105. doi: 10.1371/journal.ppat.1013105 (PMC12124754; doi:10.1371/journal.ppat.1013105)
Supplement: S1 Fig — The C-terminal conserved regions of TcPiezo1 and TcPiezo2 (TriTrypDB: TcYC6_0088320/KAF8295942 and TcYC6_0007880/KAF8281887, respectively) and mPiezo1 and mPiezo2 (GenBank accession no. NP_001344278.1 and NM_001039485, respectively) [33] were aligned with MUSCLE (https://www.ebi.ac.uk/Tools/msa/muscle) and the webserver site (http://www.bioinformatics.org/sms/index.html) [77]. Identical (black) and similar (gray) amino acid residues are shaded. The groups of similar amino acids (ILV, FWY, KRH, DE, GAS, P, C, TNQM) were used for the similarity calculation. The topology from TM34 to TM38 was derived from the structure of mPiezo1 [78]. The PFEW motif boxed in pink is conserved among plants, mammals and protozoa [7]. In mPiezo1 and mPiezo2, TM37 and TM38 are defined as outer helix (OH) and inner helix (IH) of the pore region, respectively. The C-terminal extracellular domain (CED) consists of 4 alpha domains (α1-α4) and 9 beta domains (β1-β9). The conserved residues boxed in red and green in the IH (TM38)-C-terminal domain (CTD) region form the transmembrane (TM) gate and cytosolic constriction neck of the Ca2+-transducing pore [34–36]. (PDF) [file ppat.1013105.s001.pdf]

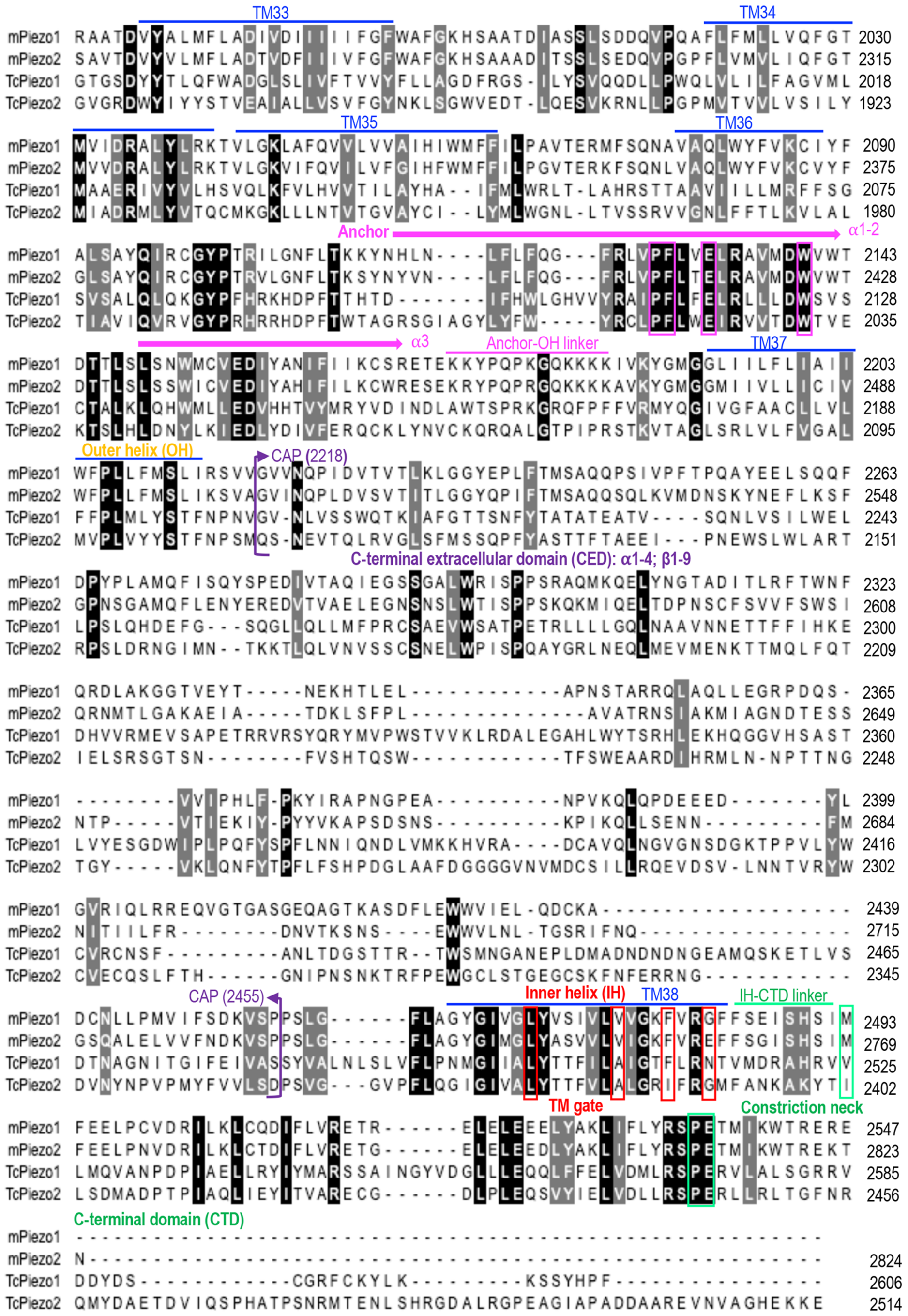

S1 Fig. Multiple sequence alignment. The C-terminal conserved regions of TcPiezo1 and TcPiezo2 (TriTrypDB: TcYC6_0088320/KAF8295942 and TcYC6_0007880/KAF8281887, respectively) and mPiezo1 and mPiezo2 (GenBank accession no. NP_001344278.1 and NM_001039485, respectively) [33] were aligned with MUSCLE (https://www.ebi.ac.uk/Tools/msa/muscle) and the webserver site (http://www.bioinformatics.org/sms/index.html) [77]. Identical (*black*) and similar (*gray*) amino acid residues are shaded. The groups of similar amino acids (ILV, FWY, KRH, DE, GAS, P, C, TNQM) were used for the similarity calculation. The topology from TM34 to TM38 was derived from the structure of mPiezo1 [78]. The PFEW motif boxed in pink is conserved among plants, mammals and protozoa [7]. In mPiezo1 and mPiezo2, TM37 and TM38 are defined as outer helix (OH) and inner helix (IH) of the pore region, respectively. The C-terminal extracellular domain (CED) consists of 4 alpha domains (α1-α4) and 9 beta domains (β1-β9). The conserved residues boxed in red and green in the IH (TM38)-C-terminal domain (CTD) region form the transmembrane (TM) gate and cytosolic constriction neck of the Ca^{2+}-transducing pore [34-36].
