# Supplementary material for: Lysosome and plasma membrane Piezo channels of Trypanosoma cruzi are essential for proliferation, differentiation and infectivity
Source: PLoS Pathog. 2025 Apr 23;21(4):e1013105. doi: 10.1371/journal.ppat.1013105 (PMC12124754; doi:10.1371/journal.ppat.1013105)
Supplement: S2 Fig — The structures of C-terminal conserved sequences (S1 Fig) of mPiezo1 (A), mPiezo2 (C), TcPiezo1 (B), and TcPiezo2 (D), were predicted with AlphaFold II [37]. A ribbon diagram of the pore region, formed by OH (TM37) and IH (TM38) of Piezo. The specific regions or domains are labelled: alpha domains (α1-α4); beta domains (β1-β12); OH, outer helix; anchor; IH, inner helix; CTD, C-terminal domain; IH-CTD linker; TM34–36. (PDF) [file ppat.1013105.s002.pdf]

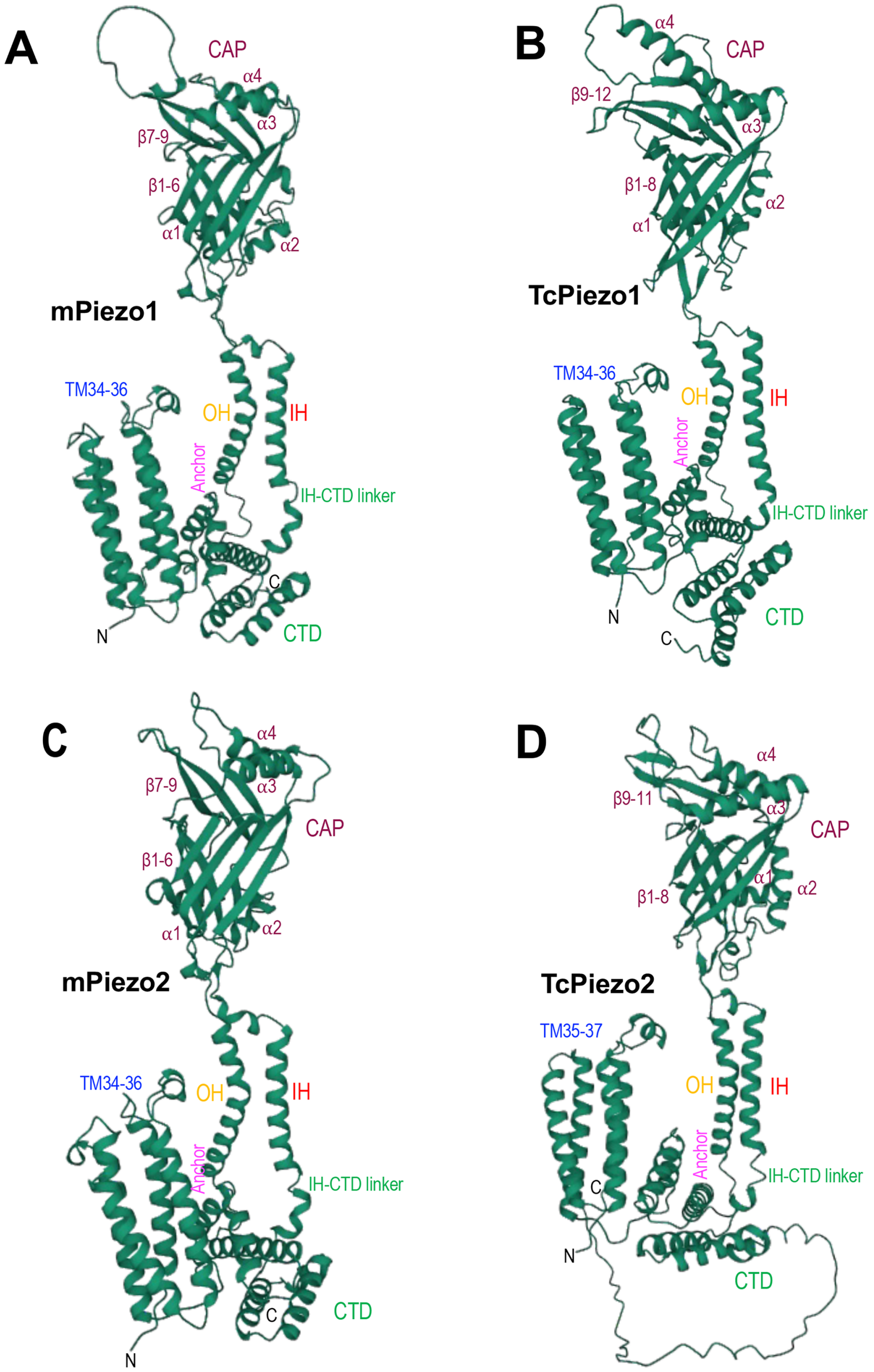


**S2 Fig. AlphaFold II structure predictions.** The structures of C-terminal conserved sequences (S1 Fig.) of mPiezo1 (A), mPiezo2 (C), TcPiezo1 (B), and TcPiezo2 (D), were predicted with AlphaFold II [37]. A ribbon diagram of the pore region, formed by OH (TM37) and IH (TM38) of Piezo. The specific regions or domains are labelled: alpha domains (α1-α4); beta domains (β1-β12); OH, outer helix; anchor; IH, inner helix; CTD, C-terminal domain; IH-CTD linker; TM34-36.
