# Supplementary material for: Lysosome and plasma membrane Piezo channels of Trypanosoma cruzi are essential for proliferation, differentiation and infectivity
Source: PLoS Pathog. 2025 Apr 23;21(4):e1013105. doi: 10.1371/journal.ppat.1013105 (PMC12124754; doi:10.1371/journal.ppat.1013105)
Supplement: S3 Fig — (A) Schematic representation of the strategy used to generate TcPiezo-KO mutants by CRISPR/Cas9-induced homologous recombination. Single guide (sg) RNAs (sgRNA1, sgRNA2, sgRNA3) were generated with Cas9 endonuclease to bind and cut the Piezo pore region (boxed) or the whole TcPiezo1 or TcPiezo2 genes. DNA was repaired or replaced with a blasticidin (bsd) or puromycin (puro) resistance gene cassette, which was generated by PCR using a set of forward and reverse primers (TcPZ-bsd-koF and TcPZ-bsd-koR, TcPZ-puro-koF and TcPZ-puro-koR, S1 Table) containing 120-bp homologous regions from the TcPiezo loci. (B) Schematic diagram of CRISPR/Cas9 mediated endogenous C-terminal tagging of TcPiezo with an aptazyme cassette (composed of 3 × Ty tag, ribozyme, GAPDH 3’UTR, and Bsd gene), which was generated by PCR with primers TcPZ-kd-F and TcPZ-kd-R (S1 Table). (C) The aptazyme cassette integrated into the C-terminal loci of TcPiezo by homologous recombination. TcPiezo tagging was verified by PCR using primers TcPZ-ORF-F and TcPZ-3UTR-R (S1 Table). The intact loci generated a PCR product of ~ 0.8 kb from parental Y strain while the tagged loci generated a fragment of ~ 1.9 kb from a homozygous cell line. (D) PCR analysis showing that both loci of TcPiezo1 were tagged with aptazyme Tet-OFF or Theo-OFF. (E) PCR analysis showing that both loci of TcPiezo2 were tagged with aptazyme Tet-OFF or Theo-OFF. (F) Illustration of the effect of tetracycline (Tet) or theophylline (Theo) binding to hammerhead ribozyme (HHR) on TcPiezo mRNA stability. (PDF) [file ppat.1013105.s003.pdf]

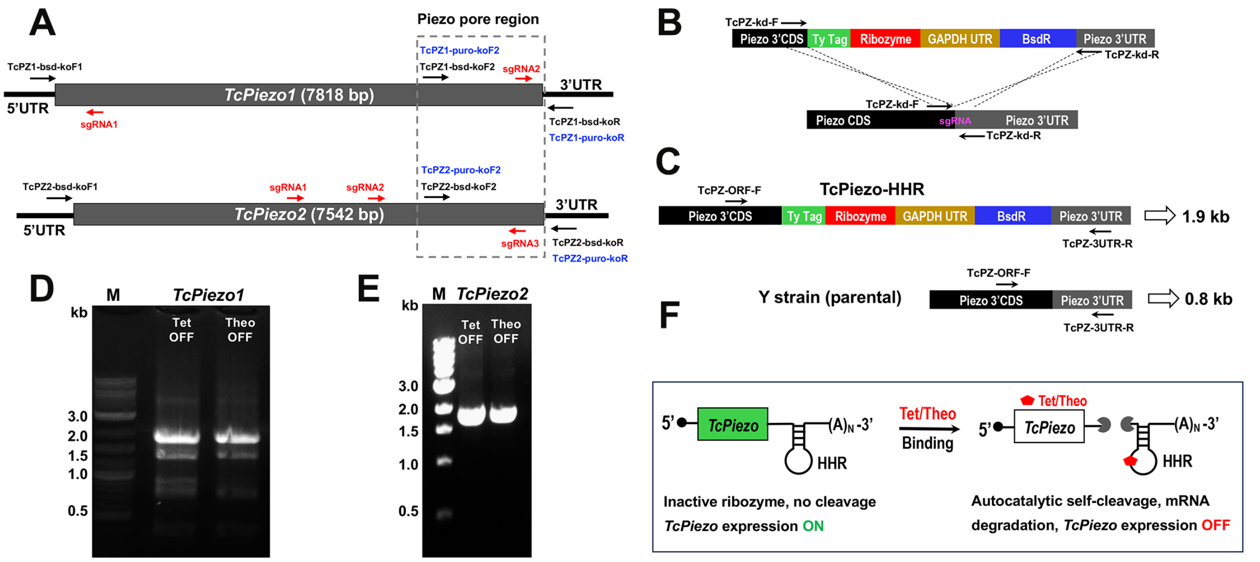

S3 Fig. Knockout (KO) and conditional KO of *TcPiezo1* or *TcPiezo2*. (A) Schematic representation of the strategy used to generate TcPiezo-KO mutants by CRISPR/Cas9-induced homologous recombination. Single guide (sg) RNAs (sgRNA1, sgRNA2, sgRNA3) were generated with Cas9 endonuclease to bind and cut the Piezo pore region (boxed) or the whole *TcPiezo1* or *TcPiezo2* genes. DNA was repaired or replaced with a blasticidin (*bsd*) or puromycin (*puro*) resistance gene cassette, which was generated by PCR using a set of forward and reverse primers (TcPZ-bsd-koF and TcPZ-bsd-koR, TcPZ-puro-koF and TcPZ-puro-koR, S1 Table) containing 120-bp homologous regions from the *TcPiezo* loci. (B) Schematic diagram of CRISPR/Cas9 mediated endogenous C-terminal tagging of *TcPiezo* with an aptazyme cassette (composed of 3×Ty tag, ribozyme, GAPDH 3'UTR, and *Bsd* gene), which was generated by PCR with primers TcPZ-kd-F and TcPZ-kd-R (S1 Table). (C) The aptazyme cassette integrated into the C-terminal loci of *TcPiezo* by homologous recombination. *TcPiezo* tagging was verified by PCR using primers TcPZ-ORF-F and TcPZ-3UTR-R (S1 Table). The intact loci generated a PCR product of ~ 0.8 kb from parental Y strain while the tagged loci generated a fragment of ~1.9 kb from a homozygous cell line. (D) PCR analysis showing that both loci of *TcPiezo1* were tagged with aptazyme *Tet-OFF* or *Theo-OFF*. (E) PCR analysis showing that both loci of *TcPiezo2* were tagged with aptazyme *Tet-OFF* or *Theo-OFF*. (F) Illustration of the effect of tetracycline (Tet) or theophylline (Theo) binding to hammerhead ribozyme (HHR) on *TcPiezo* mRNA stability.
