# Supplementary material for: Lysosome and plasma membrane Piezo channels of Trypanosoma cruzi are essential for proliferation, differentiation and infectivity
Source: PLoS Pathog. 2025 Apr 23;21(4):e1013105. doi: 10.1371/journal.ppat.1013105 (PMC12124754; doi:10.1371/journal.ppat.1013105)
Supplement: S4 Fig — (A) Western blot analyses of TcPiezo1 Tet-OFF and TcPiezo1 Theo-OFF epimastigotes grown in the absence of tetracycline (-Tet) or theophylline (-Theo). Total lysates (30 μg) were subjected to 10% SDS-polyacrylamide gel electrophoresis before transfer to a nitrocellulose membrane and then stained with antibodies against Ty1 (top). One band of ~ 290 kDa was detected in epimastigote homogenates. Membranes were stripped and re-incubated with antibody against Alpha-tubulin as a loading control (bottom, Tub). (B) Growth of TcPiezo1 Theo-OFF epimastigotes in the absence (black line, -Theo) or presence (red line, + Theo) of 250 μg ml−1 theophylline for the indicated number of days. Western blot analyses of TcPiezo1 Theo-OFF epimastigotes grown in the absence (0) or presence (2–6) of theophylline. Total lysates were subjected to 10% SDS-PAGE before transfer to a nitrocellulose membrane and stained with Ab against Ty1. Bands of 290 kDa were detected. Alpha-tubulin was used as loading control. (C) Percentage of metacyclic trypomastigotes in TcPiezo1 Theo-OFF epimastigote cultures after incubation in TAU 3AAG medium in the absence (-Theo) and presence (+Theo) of 250 µ g/ml theophylline after 96 h. (D) Effect of non-induced (-Theo) and induced (+Theo) TcPiezo1 Theo-OFF on trypomastigote infection of Vero cells after 4 h. (E) Effect of non-induced (-Theo) and induced (+Theo) TcPiezo1 Theo-OFF on amastigote replication after 72 h. In panels B, C, D, E, values are mean ± s.d. (n = 3). One-way ANOVA with multiple comparisons (*P < 0.05, **P < 0.01, ***P < 0.001). (F) Representative images of Vero cell infected with Tet-induced (+Tet) and non-induced (-Tet) TcPiezo1 Tet-OFF trypomastigotes. (G) Representative images of Vero cell infected with Theo-induced (+Theo) and non-induced (-Theo) TcPiezo1 Theo-OFF trypomastigotes. In panels F and G, nuclei and kinetoplasts were DAPI stained. Scale bars 10 µm. (PDF) [file ppat.1013105.s004.pdf]

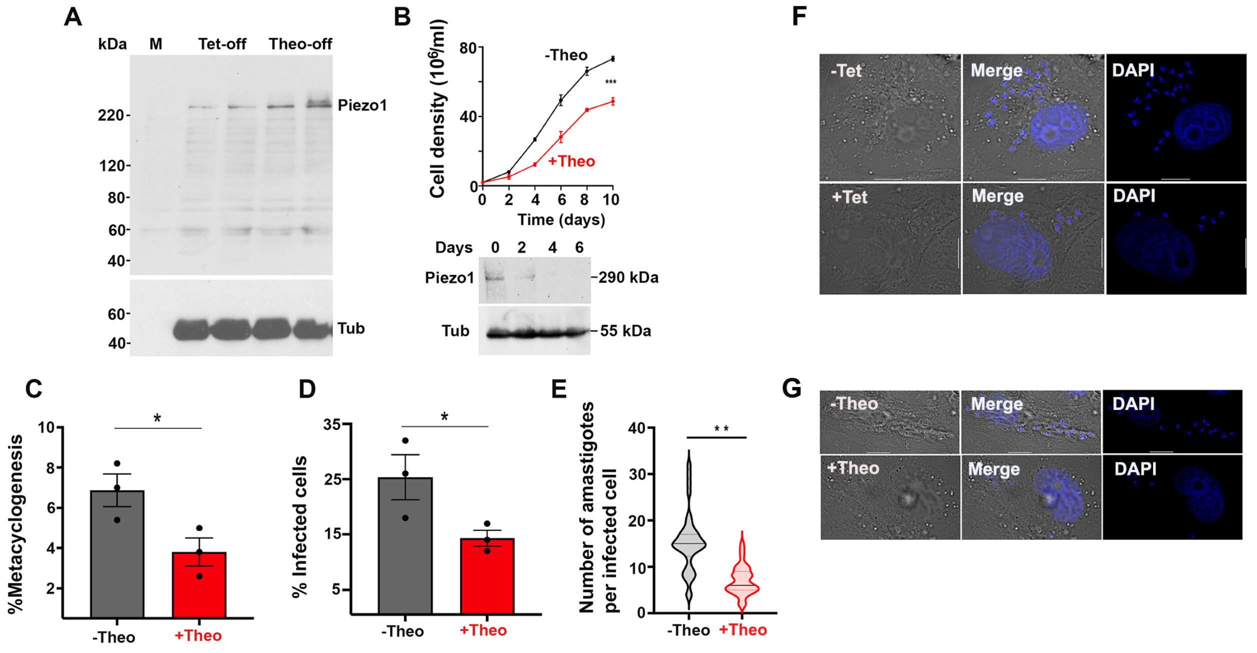

S4 Fig. Characterization of *TcPiezo1-CKO*. (A) Western blot analyses of *TcPiezo1 Tet-OFF* and *TcPiezo1 Theo-OFF* epimastigotes grown in the absence of tetracycline (-Tet) or theophylline (-Theo). Total lysates (30 µg) were subjected to 10% SDS-polyacrylamide gel electrophoresis before transfer to a nitrocellulose membrane and then stained with antibodies against Ty1 (top). One band of ~290 kDa was detected in epimastigote homogenates. Membranes were stripped and re-incubated with antibody against Alpha-tubulin as a loading control (bottom, Tub). (B) Growth of *TcPiezo1 Theo-OFF* epimastigotes in the absence (black line, *-Theo*) or presence (red line, *+Theo*) of 250 µg ml^{-1} theophylline for the indicated number of days. Western blot analyses of *TcPiezo1 Theo-OFF* epimastigotes grown in the absence (0) or presence (2–6) of theophylline. Total lysates were subjected to 10% SDS-PAGE before transfer to a nitrocellulose membrane and stained with Ab against Ty1. Bands of 290 kDa were detected. Alpha-tubulin was used as loading control. (C) Percentage of metacyclic trypomastigotes in *TcPiezo1 Theo-OFF* epimastigote cultures after incubation in TAU 3AAG medium in the absence (-Theo) and presence (+Theo) of 250 µg/ml theophylline after 96 h. (D) Effect of non-induced (-Theo) and induced (+Theo) *TcPiezo1 Theo-OFF* on trypomastigote infection of Vero cells after 4 h. (E) Effect of non-induced (-Theo) and induced (+Theo) *TcPiezo1 Theo-OFF* on amastigote replication after 72 h. In panels B, C, D, E, values are mean ± s.d. (n=3). One-way ANOVA with multiple comparisons ($*P < 0.05$, $**P < 0.01$, $***P < 0.001$). (F) Representative images of Vero cell infected with Tet-induced (+Tet) and non-induced (-Tet) *TcPiezo1 Tet-OFF* trypomastigotes. (G) Representative images of Vero cell infected with Theo-induced (+Theo) and non-induced (-Theo) *TcPiezo1 Theo-OFF* trypomastigotes. In panels F and G, nuclei and kinetoplasts were DAPI stained. Scale bars 10 µm**.**
