# Supplementary material for: Lysosome and plasma membrane Piezo channels of Trypanosoma cruzi are essential for proliferation, differentiation and infectivity
Source: PLoS Pathog. 2025 Apr 23;21(4):e1013105. doi: 10.1371/journal.ppat.1013105 (PMC12124754; doi:10.1371/journal.ppat.1013105)
Supplement: S5 Fig — (A, B) Ca2+ entry was affected by TcPiezo1 downregulation in T. cruzi amastigotes. Fura-2/AM loaded induced (+Tet/ + Theo) or uninduced (-Tet/-Theo) amastigotes were suspended in buffer with 100 µ M EGTA and 1.8 mM CaCl2 were added at 150 sec (arrows). Cytosolic Ca2+ concentrations in the tissue-derived amastigotes were quantified in nM. (C, D) Intracellular Ca2+ by TcPiezo1 Theo-OFF epimastigotes (C) or trypomastigotes (D) expressing jGCaMP7s in AU. (C) Addition of 1.8 mM Ca2+ in Theo-induced TcPiezo1 cells (+Theo) elicited a lower increase in intracellular Ca2+ than in non-induced cells (-Theo). (D) ECM triggered TcPiezo1-mediated Ca2+ entry in trypomastigotes. Addition of 40 µg ECM to non-induced (-Theo) trypomastigotes significantly increased intracellular Ca2+, compared to Theo-induced cells (+Theo). In panels C-D, addition of BAG (baseline, B), instead of 1.8 mM Ca2+, was used as a control. Values are means ± s.d. (n = 3). One-way ANOVA with multiple comparisons (* P < 0.05, **P < 0.01, ***P < 0.001). (PDF) [file ppat.1013105.s005.pdf]

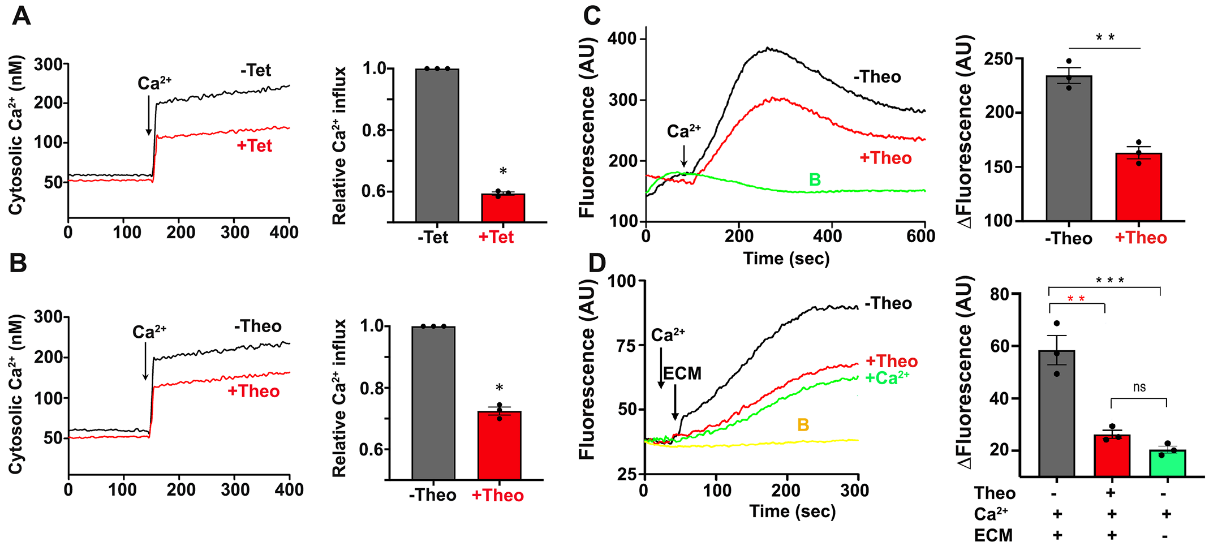

S5 Fig. Downregulation of *TcPiezo1* expression reduces Ca^{2+} entry of *T. cruzi*. (A, B) Ca^{2+} entry was affected by *TcPiezo1* downregulation in *T. cruzi* amastigotes. Fura-2/AM loaded induced (+Tet/+Theo) or uninduced (-Tet/-Theo) amastigotes were suspended in buffer with 100 µM EGTA and 1.8 mM $CaCl_2$ were added at 150 sec (arrows). Cytosolic Ca^{2+} concentrations in the tissue-derived amastigotes were quantified in nM. (C, D) Intracellular Ca^{2+} by *TcPiezo1 Theo-OFF* epimastigotes (C) or trypomastigotes (D) expressing jGCaMP7s in AU. (C) Addition of 1.8 mM Ca^{2+} in Theo-induced *TcPiezo1* cells (+Theo) elicited a lower increase in intracellular Ca^{2+} than in non-induced cells (-Theo). (D) ECM triggered TcPiezo1-mediated Ca^{2+} entry in trypomastigotes. Addition of 40 µg ECM to non-induced (-Theo) trypomastigotes significantly increased intracellular Ca^{2+}, compared to Theo-induced cells (+Theo). In panels C-D, addition of BAG (baseline, B), instead of 1.8 mM Ca^{2+}, was used as a control. Values are means ± s.d. (n=3). One-way ANOVA with multiple comparisons (* $P < 0.05$, ** $P < 0.01$, *** $P < 0.001$).
