# Supplementary material for: Lysosome and plasma membrane Piezo channels of Trypanosoma cruzi are essential for proliferation, differentiation and infectivity
Source: PLoS Pathog. 2025 Apr 23;21(4):e1013105. doi: 10.1371/journal.ppat.1013105 (PMC12124754; doi:10.1371/journal.ppat.1013105)
Supplement: S6 Fig — (A-C) Expression of GCaMP Ca2+ sensors in T. cruzi WT. (A) Western blot analysis of lysates labels a band of 50 kDa with antibody against GFP. Alpha-tubulin was used as loading control. (B) Representative traces of epimastigotes expressing GCaMP6f (6f), GCaMP6s (6s), jGCaMP7f (7f), and jGCaMP7s (7s) incubated in BAG, showing extracellular and intracellular Ca2+ responses to 1 µ M ionomycin. (C) Fixed epimastigotes expressing GCaMP Ca2+ sensors (GCaMP6f, GCaMP6s, jGCaMP7f, and jGCaMP7s) assayed by immunofluorescence microscopy with antibody against GFP showing cytosolic localization. Scale bars 10 µm. (D-I) Expression of jGCaMP7s in TcPiezo-CKO epimastigotes (TcPiezo1 Tet-OFF/Theo-OFF (D-F) and TcPiezo2 Tet-OFF/Theo-OFF (G-I). (D, G) TcPiezo Tet-OFF/Theo-OFF epimastigotes expressing jGCaMP7s assayed by immunofluorescence microscopy with antibody against His showing cytosolic localization. DIC, differential interference contrast microscopy. Scale bars 10 µm. (E, H) Western blot analysis of TcPiezo-CKO lysates labels a band of 50 kDa with antibody against His. Alpha-tubulin was used as loading control. (F, I) Representative traces of TcPiezo-CKO epimastigotes expressing jGCaMP7s incubated in BAG, showing intracellular Ca2+ responses to 1 µ M ionomycin. 100 µ M EGTA was added to remove extracellular Ca2+. (PDF) [file ppat.1013105.s006.pdf]

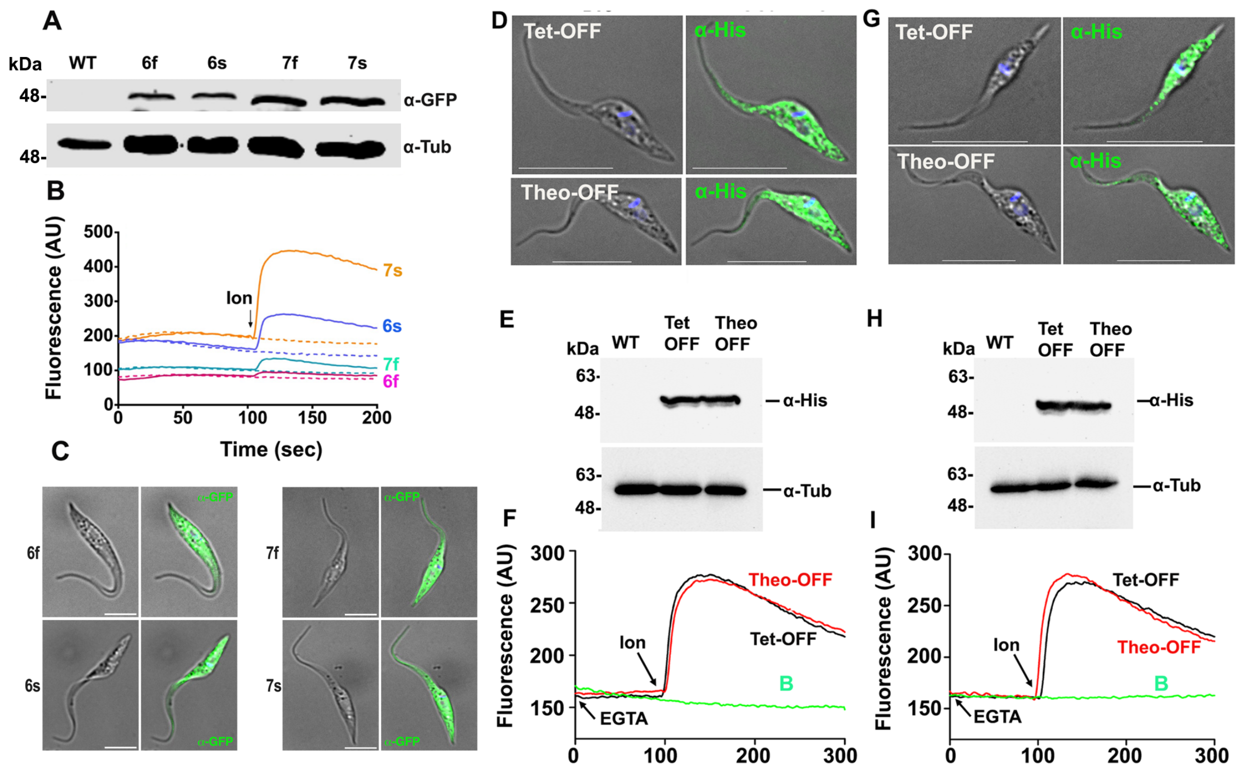

S6 Fig. Expression of GCaMP Ca^{2+} sensors in *T. cruzi.* (A-C) Expression of GCaMP Ca^{2+} sensors in *T. cruzi* WT. (A) Western blot analysis of lysates labels a band of 50 kDa with antibody against GFP. Alpha-tubulin was used as loading control. (B) Representative traces of epimastigotes expressing GCaMP6f (6f), GCaMP6s (6s), jGCaMP7f (7f), and jGCaMP7s (7s) incubated in BAG, showing extracellular and intracellular Ca^{2+} responses to 1 µM ionomycin. (C) Fixed epimastigotes expressing GCaMP Ca^{2+} sensors (GCaMP6f, GCaMP6s, jGCaMP7f, and jGCaMP7s) assayed by immunofluorescence microscopy with antibody against GFP showing cytosolic localization. Scale bars 10 µm. (D-I) Expression of jGCaMP7s in *TcPiezo-CKO* epimastigotes (*TcPiezo1 Tet-OFF/Theo-OFF* (D-F) and *TcPiezo2 Tet-OFF/Theo-OFF* (G-I). (D, G) *TcPiezo Tet-OFF/Theo-OFF* epimastigotes expressing jGCaMP7s assayed by immunofluorescence microscopy with antibody against His showing cytosolic localization. DIC, differential interference contrast microscopy. Scale bars 10 µm. (E, H) Western blot analysis of *TcPiezo-CKO* lysates labels a band of 50 kDa with antibody against His. Alpha-tubulin was used as loading control. (F, I) Representative traces of *TcPiezo-CKO* epimastigotes expressing jGCaMP7s incubated in BAG, showing intracellular Ca^{2+} responses to 1 µM ionomycin. 100 µM EGTA was added to remove extracellular Ca^{2+}.
