# Supplementary material for: Lysosome and plasma membrane Piezo channels of Trypanosoma cruzi are essential for proliferation, differentiation and infectivity
Source: PLoS Pathog. 2025 Apr 23;21(4):e1013105. doi: 10.1371/journal.ppat.1013105 (PMC12124754; doi:10.1371/journal.ppat.1013105)
Supplement: S7 Fig — (A) smV5-tagged TcPiezo2 colocalized with T. cruzi serine carboxypeptidase (TcSC) to the reservosomes/lysosomes of epimastigotes (PCC: 0.7024), trypomastigotes (PCC: 0.8293) and amastigotes (PCC: 0.7933). Yellow in merged images indicates colocalization. DIC, differential interference contrast microscopy. Scale bars 5 or 10 µm, as indicated. (B) Western blot analysis of C-terminally smV5-tagged TcPiezo2 epimastigotes. Total lysates (30 μg) were subjected to 10% SDS-polyacrylamide gel electrophoresis before transfer to a nitrocellulose membrane and then stained with antibodies against V5 (top). One band of ~ 320 kDa was detected in epimastigote homogenates. Membranes were stripped and re-incubated with antibody against Alpha-tubulin as a loading control (bottom). (C) Growth of TcPiezo2 Theo-OFF epimastigotes in the absence (black line, − Theo) or presence (red line, + Theo) of 250 μg ml−1 theophylline for the indicated number of days. Western blot analyses of TcPiezo2 Theo-OFF epimastigotes grown in the absence (0) or presence (2–6) of theophylline. Total lysates were subjected to 10% SDS-PAGE before transfer to a nitrocellulose membrane and stained with Ab against Ty1. Bands of 280 kDa were detected. Alpha-tubulin was used as loading control. (D) Percentage of metacyclic trypomastigotes in TcPiezo2 Theo-OFF epimastigote cultures after incubation in TAU 3AAG medium in the absence (-Theo) and presence (+Theo) of 250 µ g/ml theophylline after 96 h. (E) Effect of non-induced (-Theo) and induced (+Theo) TcPiezo2 Theo-OFF on trypomastigote infection of Vero cells after 4 h. (F) Effect of non-induced (-Theo) and induced (+Theo) TcPiezo2-CKO on amastigote replication after 72 h. In panels C, D, E, F, values are mean ± s.d. (n = 3). One-way ANOVA with multiple comparisons (**P < 0.01, ***P < 0.001). (G) Representative images of Vero cell infected with Tet-induced (+Tet) and non-induced (-Tet) TcPiezo2 Tet-OFF trypomastigotes. (H) Representative images of Vero cell infected w [file ppat.1013105.s007.pdf]

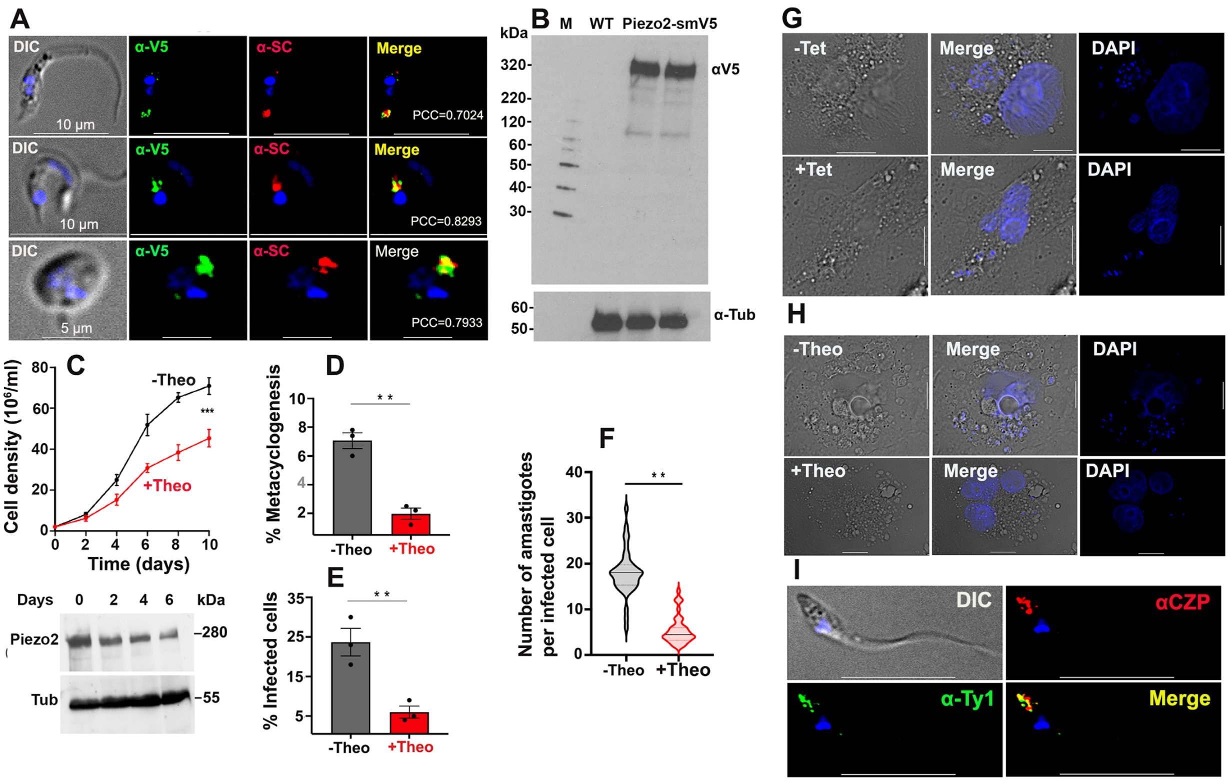

S7 Fig. Subcellular localization and characterization of *TcPiezo2 Theo-OFF*. (A) smV5-tagged TcPiezo2 colocalized with *T. cruzi* serine carboxypeptidase (TcSC) to the reservosomes/lysosomes of epimastigotes (PCC: 0.7024), trypomastigotes (PCC: 0.8293) and amastigotes (PCC: 0.7933). Yellow in merged images indicates colocalization. DIC, differential interference contrast microscopy. Scale bars 5 or 10 µm, as indicated. (B) Western blot analysis of C-terminally smV5-tagged TcPiezo2 epimastigotes. Total lysates (30 µg) were subjected to 10% SDS-polyacrylamide gel electrophoresis before transfer to a nitrocellulose membrane and then stained with antibodies against V5 (top). One band of ~320 kDa was detected in epimastigote homogenates. Membranes were stripped and re-incubated with antibody against Alpha-tubulin as a loading control (bottom). (C) Growth of *TcPiezo2 Theo-OFF* epimastigotes in the absence (black line, −*Theo*) or presence (red line, + *Theo*) of 250 µg ml^{-1} theophylline for the indicated number of days. Western blot analyses of *TcPiezo2 Theo-OFF* epimastigotes grown in the absence (0) or presence (2–6) of theophylline. Total lysates were subjected to 10% SDS-PAGE before transfer to a nitrocellulose membrane and stained with Ab against Ty1. Bands of 280 kDa were detected. Alpha-tubulin was used as loading control. (D) Percentage of metacyclic trypomastigotes in *TcPiezo2 Theo-OFF* epimastigote cultures after incubation in TAU 3AAG medium in the absence (-*Theo*) and presence (+*Theo*) of 250 µg/ml theophylline after 96 h. (E) Effect of non-induced (-*Theo*) and induced (+*Theo*) *TcPiezo2 Theo-OFF* on trypomastigote infection of Vero cells after 4 h. (F) Effect of non-induced (-*Theo*) and induced (+*Theo*) *TcPiezo2-CKO* on amastigote replication after 72 h. In panels C, D, E, F, values are mean ± s.d. (n=3). One-way ANOVA with multiple comparisons (**$P < 0.01$, ***$P < 0.001$). (G) Representative images of Vero cell infected with Tet-induced (+Tet) and non-induced (-Tet) *TcPiezo2 Tet-OFF* trypomastigotes. (H) Representative images of Vero cell infected with Theo-induced (+Theo) and non-induced (-Theo) *TcPiezo2 Theo-OFF* trypomastigotes. In panels G and H, nuclei and kinetoplasts were DAPI stained. Scale bars 10 µm. (I) Ty1-tagged TcPiezo2 Tet-OFF colocalization with *T. cruzi* cruzipain (TcCZP) to the reservosomes/lysosomes of epimastigotes (PCC: 0.7892), as detected by immunofluorescence analyses with antibodies against Ty1. Yellow in merged images indicates colocalization. DIC, differential interference contrast microscopy. Scale bars 10 µm.
