# Supplementary material for: Lysosome and plasma membrane Piezo channels of Trypanosoma cruzi are essential for proliferation, differentiation and infectivity
Source: PLoS Pathog. 2025 Apr 23;21(4):e1013105. doi: 10.1371/journal.ppat.1013105 (PMC12124754; doi:10.1371/journal.ppat.1013105)
Supplement: S8 Fig — Intracellular Ca2+ concentrations of 5 × 10^7^ TcPiezo2 Theo-OFF epimastigotes expressing jGCaMP7s were measured by jGCaMP7s signal fluorescence in AU. (A, B) 100 µ M EGTA was incubated with TcPiezo2 Theo-OFF cells to remove extracellular Ca2+, abolishing TcPiezo1-mediated Ca2+ entry. (A) TcPiezo2 mediated lysosomal Ca2+ release. Addition of 70 µ M GPN for activation of lysosomal Ca2+ release in Theo-induced TcPiezo2 cells (+Theo) elicited a lower increase in intracellular Ca2+ than in non-induced cells (-Theo).(B) Addition of 30 µ M CPA for activation of ER Ca2+ release demonstrated no significant difference between Theo-induced (+Theo) and non-induced (-Theo) cells. (C) Addition of 1.8 mM Ca2+ showed no significant difference in intracellular Ca2+ between Theo-induced (+Theo) and non-induced (-Theo) cells. (D) 100 µ M EGTA was added to remove extracellular Ca2+. Addition of 1 µ M ionomycin in Theo-induced cells (+Theo) elicited less increase of intracellular Ca2+ than in non-induced cells (-Theo). Addition of DMSO or BAG was used as control (baseline) labeled with B. Values are means ± s.d. (n = 3). One-way ANOVA with multiple comparisons (*P < 0.05). (PDF) [file ppat.1013105.s008.pdf]

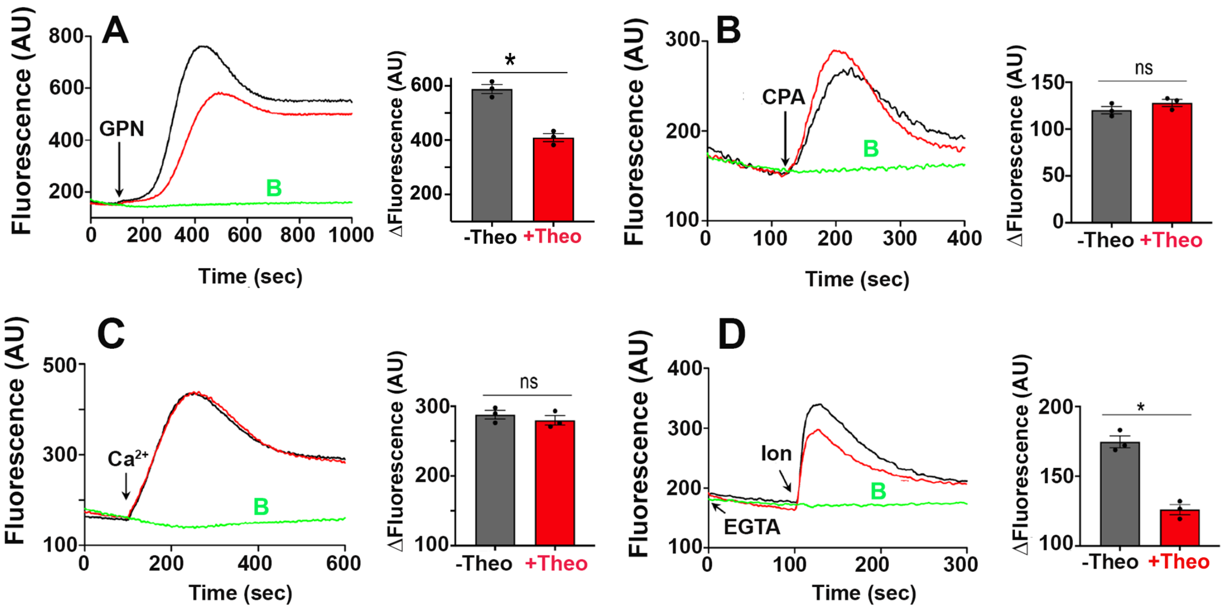

S8 Fig. Effects of *TcPiezo2* expression downregulation on intracellular Ca^{2+} stores in *T. cruzi*. Intracellular Ca^{2+} concentrations of 5×10^7 *TcPiezo2 Theo-OFF* epimastigotes expressing jGCaMP7s were measured by jGCaMP7s signal fluorescence in AU. (A, B) 100 µM EGTA was incubated with *TcPiezo2 Theo-OFF* cells to remove extracellular Ca^{2+}, abolishing TcPiezo1-mediated Ca^{2+} entry. (A) TcPiezo2 mediated lysosomal Ca^{2+} release. Addition of 70 µM GPN for activation of lysosomal Ca^{2+} release in Theo-induced *TcPiezo2* cells (+Theo) elicited a lower increase in intracellular Ca^{2+} than in non-induced cells (-Theo).(B) Addition of 30 µM CPA for activation of ER Ca^{2+} release demonstrated no significant difference between Theo-induced (+Theo) and non-induced (-Theo) cells. (C) Addition of 1.8 mM Ca^{2+} showed no significant difference in intracellular Ca^{2+} between Theo-induced (+Theo) and non-induced (-Theo) cells. (D) 100 µM EGTA was added to remove extracellular Ca^{2+}. Addition of 1 µM ionomycin in Theo-induced cells (+Theo) elicited less increase of intracellular Ca^{2+} than in non-induced cells (-Theo). Addition of DMSO or BAG was used as control (baseline) labeled with B. Values are means ± s.d. (n=3). One-way ANOVA with multiple comparisons ($*P < 0.05$).
