# Supplementary material for: Lysosome and plasma membrane Piezo channels of Trypanosoma cruzi are essential for proliferation, differentiation and infectivity
Source: PLoS Pathog. 2025 Apr 23;21(4):e1013105. doi: 10.1371/journal.ppat.1013105 (PMC12124754; doi:10.1371/journal.ppat.1013105)
Supplement: S9 Fig — Intracellular Ca2+ changes of 5 × 10^7^ TcPiezo Theo-OFF epimastigotes expressing jGCaMP7s were measured by jGCaMP7s signal fluorescence in AU. (A) Dooku1-evoked TcPiezo1-mediated Ca2+ entry. Two Ca2+ spikes were generated in Theo-induced (+Theo) and non-induced (-Theo) TcPiezo1 Theo-OFF cells, respectively, by the additions of 1.8 mM Ca2+ and 40 µ M Dooku1. Changes in jGCaMP7s fluorescence (first peak and second peak) in Theo induced (+Theo) and non-induced (-Theo) cells were shown in bar graphs. Downregulation of TcPiezo1 expression significantly decreased Dooku1-evoked TcPiezo1-mediated Ca2+ entry (left bar graph) but had no effect on Dooku1-evoked TcPiezo2-mediated Ca2+ release (right bar graph). (B) 100 µ M EGTA was incubated with TcPiezo2 Theo-OFF cells to remove extracellular Ca2+, abolishing TcPiezo1-mediated Ca2+ entry. 70µ M GPN was added to activate Ca2+ release from lysosomes in the cells. Downregulation of TcPiezo2 expression (+Theo) showed a significant decrease of lysosomal Ca2+ release as in S8A Fig. Addition of 10 µ M GsMTx4 showed a significant reduction of intracellular Ca2+ in non-induced cells (-Theo+GsM) like downregulation of TcPiezo2 (+Theo) and had an additive effect on TcPiezo2-mediated Ca2+ release in Theo-induced cells (+Theo+GsM). Changes in jGCaMP7s fluorescence and the rates of fluorescence increase upon GsM inhibition in Theo-induced (+Theo±GsM) and non-induced (-Theo±GsM) cells were shown in left and right bar graphs, respectively. (C) 100 µ M EGTA and 70 µ M GPN were added to TcPiezo2 Theo-OFF cells (±Theo), as indicated. Addition of 20 µ M Yoda1 (+Yoda1) dramatically stimulated TcPiezo2-mediated Ca2+ release from lysosomes. Changes in jGCaMP7s fluorescence and the rates of fluorescence increase upon Yoda1 activation in Tet-induced (+Tet±Yoda1) and non-induced (-Te±Yoda1) cells were shown in left and right bar graphs, respectively. (D) Jedi 1 and Jedi2 did not activate TcPiezo1. Additions of 3 pulses of 200 µ M Jedi1 or Jedi2 at 100 s [file ppat.1013105.s009.pdf]

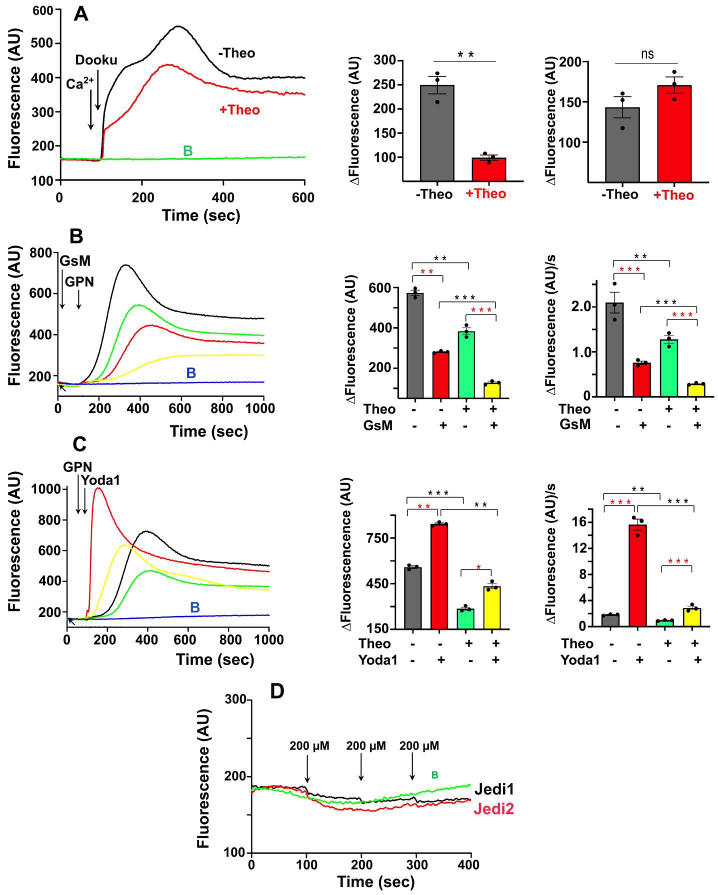


**S9 Fig. Effects of Piezo activators and inhibitors on TcPiezo channels.** Intracellular $Ca^{2+}$ changes of 5 × $10^7$ *TcPiezo Theo-OFF* epimastigotes expressing jGCaMP7s were measured by jGCaMP7s signal fluorescence in AU. (A) Dooku1-evoked TcPiezo1-mediated $Ca^{2+}$ entry. Two $Ca^{2+}$ spikes were generated in Theo-induced (+Theo) and non-induced (-Theo) *TcPiezo1 Theo-OFF* cells, respectively, by the additions of 1.8 mM $Ca^{2+}$ and 40 µM Dooku1. Changes in jGCaMP7s fluorescence (first peak and second peak) in Theo induced (+Theo) and non-induced (-Theo) cells were shown in bar graphs. Downregulation of *TcPiezo1* expression significantly decreased Dooku1-evoked TcPiezo1-mediated $Ca^{2+}$ entry (left bar graph) but had no effect on Dooku1-evoked TcPiezo2-mediated $Ca^{2+}$ release (right bar graph). (B) 100 µM EGTA was incubated with *TcPiezo2 Theo-OFF* cells to remove extracellular $Ca^{2+}$, abolishing TcPiezo1-mediated $Ca^{2+}$ entry. 100 µM GPN was added to activate $Ca^{2+}$ release from lysosomes in the cells. Downregulation of *TcPiezo2* expression (+Theo) showed a significant decrease of lysosomal $Ca^{2+}$ release as in S8A Fig. Addition of 10 µM GsMTx4 showed a significant reduction of intracellular $Ca^{2+}$ in non-induced cells (-Theo+GsM) like downregulation of *TcPiezo2* (+Theo) and had an additive effect on TcPiezo2-mediated $Ca^{2+}$ release in Theo-induced cells (+Theo+GsM). Changes in jGCaMP7s fluorescence and the rates of fluorescence increase upon GsM inhibition in Theo-induced (+Theo±GsM) and non-induced (-Theo±GsM) cells were shown in left and right bar graphs, respectively. (C) 100 µM EGTA and 70 µM GPN were added to *TcPiezo2 Theo-OFF* cells (±Theo), as indicated. Addition of 20 µM Yoda1 (+Yoda1) dramatically stimulated TcPiezo2-mediated $Ca^{2+}$ release from lysosomes. Changes in jGCaMP7s fluorescence and the rates of fluorescence increase upon Yoda1 activation in Tet-induced (+Tet±Yoda1) and non-induced (-Te±Yoda1) cells were shown in left and right bar graphs, respectively. (D) Jedi 1 and Jedi2 did not activate TcPiezo1. Additions of 3 pulses of 200 µM Jedi1 or Jedi2 at 100 s, 200 s and 300 s (arrows), respectively, to non-induced *TcPiezo1 Tet-OFF* epimastigotes (5 × $10^7$ cells) did not affect intracellular $Ca^{2+}$ in the cells. Addition of DMSO, instead of Yoda1/Dooku1/GsM/Jedi1/Jedi2, was used as control (baseline) labeled with B. In bar graphs A, B, C, values are means ± s.d. ($n$=3). One-way ANOVA with multiple comparisons (**$P < 0.01$, ***$P < 0.001$).
