# Supplementary material for: Lysosome and plasma membrane Piezo channels of Trypanosoma cruzi are essential for proliferation, differentiation and infectivity
Source: PLoS Pathog. 2025 Apr 23;21(4):e1013105. doi: 10.1371/journal.ppat.1013105 (PMC12124754; doi:10.1371/journal.ppat.1013105)
Supplement: S10 Fig — TcPiezo Tet-OFF epimastigotes expressing jGCaMP7s (5 × 10^7^ cells) in 600 µl isosmotic buffer (300 mOsm) were submitted to hyperosmotic stress (800 mOsm) by adding 600 µl mannitol (1,300 mOsm) at 50 s. Under the hyperosmotic stress, the TcPiezo1 Tet-OFF (A) and TcPiezo2 Tet-OFF (B) cells shrank but no intracellular Ca2+ rose (red tracings). No intracellular Ca2+ was increased by adding 600 µl isosmotic BAG. (PDF) [file ppat.1013105.s010.pdf]

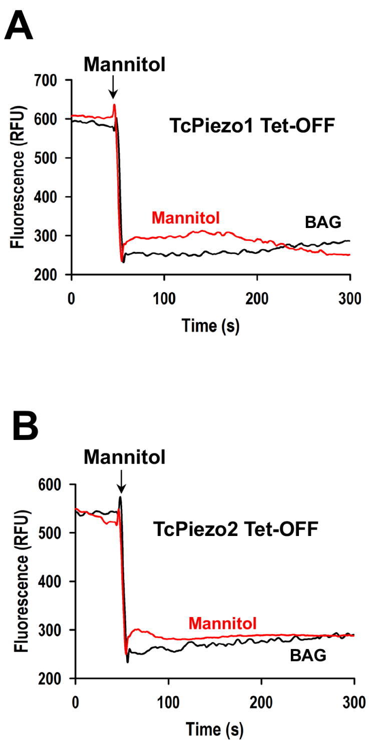

S10 Fig. *TcPiezo-CKO* responses to hyperosmotic stress. *TcPiezo Tet-OFF* epimastigotes expressing jGCaMP7s (5×10^7 cells) in 600 µl isosmotic buffer (300 mOsm) were submitted to hyperosmotic stress (800 mOsm) by adding 600 µl mannitol (1,300 mOsm) at 50 s. Under the hyperosmotic stress, the *TcPiezo1 Tet-OFF* (A) and *TcPiezo2 Tet-OFF* (B) cells shrank but no intracellular Ca^{2+} rose (red tracings). No intracellular Ca^{2+} was increased by adding 600 µl isosmotic BAG.
