# Supplementary material for: Lysosome and plasma membrane Piezo channels of Trypanosoma cruzi are essential for proliferation, differentiation and infectivity
Source: PLoS Pathog. 2025 Apr 23;21(4):e1013105. doi: 10.1371/journal.ppat.1013105 (PMC12124754; doi:10.1371/journal.ppat.1013105)
Supplement: S11 Fig — Total lysates (30 μg) were subjected to 10% SDS-polyacrylamide gel electrophoresis before transfer to a nitrocellulose membrane and then stained with antibodies against Ty1 (top). One band of ~ 290 kDa (A) or ~ 280 kD (B) was detected in trypomastigotes (Trypo) and amastigotes (Ama) homogenates. Membranes were stripped and re-incubated with antibody against Alpha-tubulin as a loading control (bottom). (PDF) [file ppat.1013105.s011.pdf]

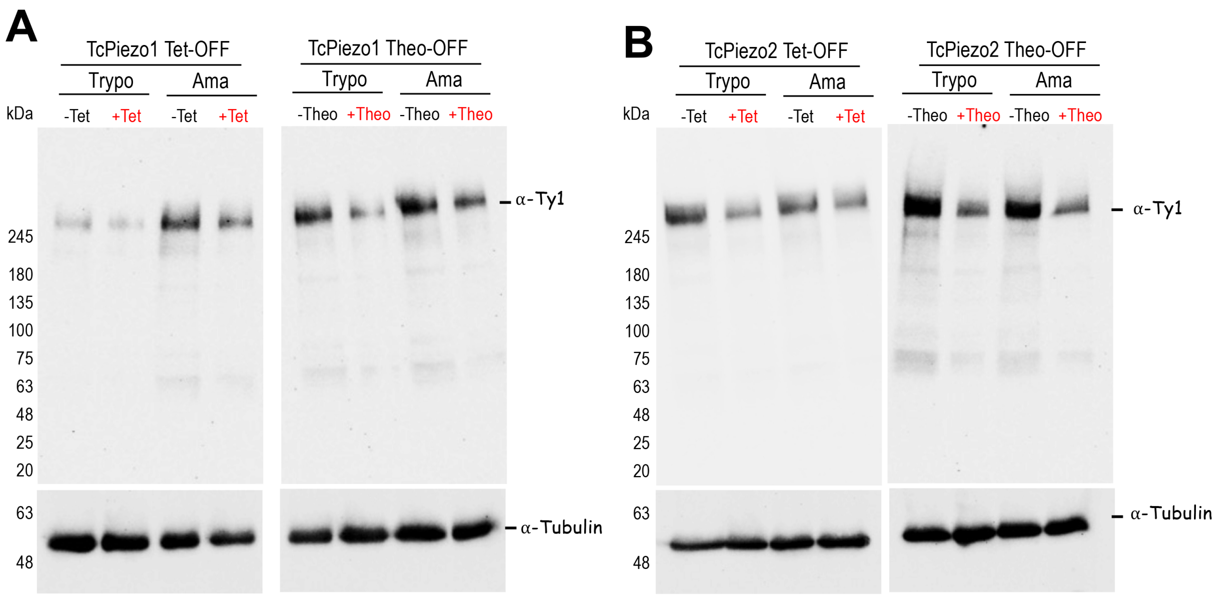


**S11 Fig. Western blot analysis of *TcPiezo Tet-OFF/Theo-OFF* trypomastigotes and amastigotes in the absence (-Tet/-Theo) or presence (+Tet/+Theo) of tetracycline or theophylline for 2 days.** Total lysates (30 µg) were subjected to 10% SDS-polyacrylamide gel electrophoresis before transfer to a nitrocellulose membrane and then stained with antibodies against Ty1 (top). One band of ~290 kDa (A) or ~280 kD (B) was detected in trypomastigotes (Trypo) and amastigotes (Ama) homogenates. Membranes were stripped and re-incubated with antibody against Alpha-tubulin as a loading control (bottom).
