# Supplementary figures and images for: Lysosome and plasma membrane Piezo channels of Trypanosoma cruzi are essential for proliferation, differentiation and infectivity
Source: PLoS Pathog. 2025 Apr 23;21(4):e1013105. doi: 10.1371/journal.ppat.1013105 (PMC12124754; doi:10.1371/journal.ppat.1013105)

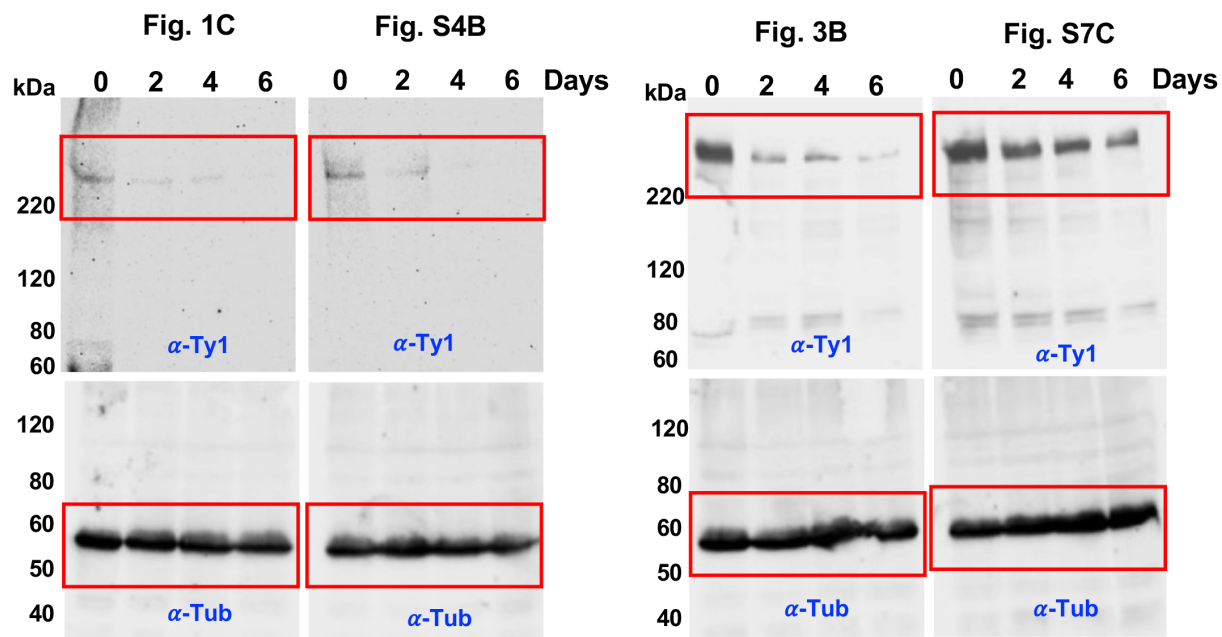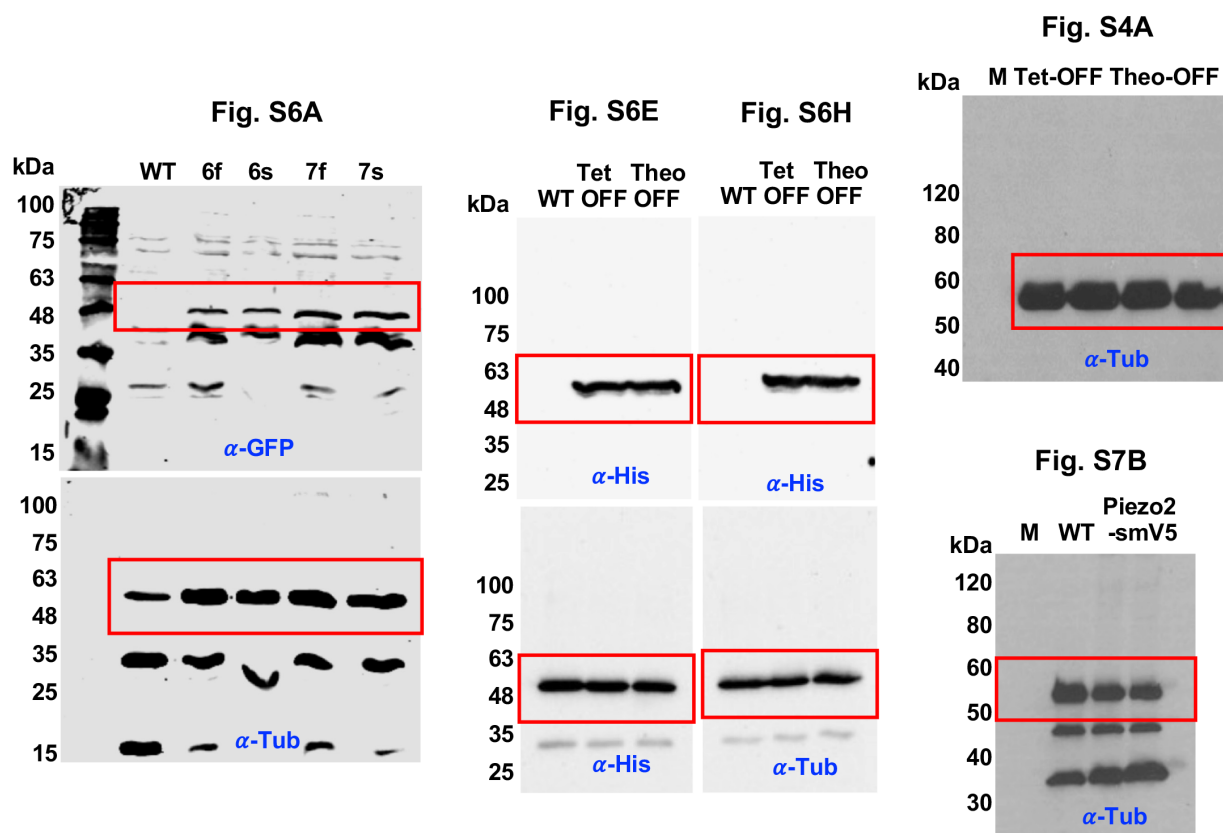

S12 Fig. Full blots shown in the article

Supplement: S12 Fig — (PDF) [file ppat.1013105.s012.pdf]
