# Supplementary material for: Lysosome and plasma membrane Piezo channels of Trypanosoma cruzi are essential for proliferation, differentiation and infectivity
Source: PLoS Pathog. 2025 Apr 23;21(4):e1013105. doi: 10.1371/journal.ppat.1013105 (PMC12124754; doi:10.1371/journal.ppat.1013105)
Supplement: S1 Table — (PDF) [file ppat.1013105.s013.pdf]

S1 Table. Primers used in this study

Name	Usage	Primer sequence (5' to 3')	Enzyme
TcPZ1-bsd-koF1	KO	AAAGTGTCACTGCTTTCATTTCTTCTTCCTTCTCAACTGCTGTTGTACGTGAGTCTGGTTTCTCCTGTCTACAGTTGTTTCTTGCCGCCACCCTTGGTTGGTTGCTTGGTCTATCATTCATGGCCAAGCCTTTGTCTCAAG	
TcPZ1-bsd-koF2	KO	ATAATAGTAACCATGATGGAATTGGTGAGTACAACAACTCAATGCGTCAAAAATGGCAAAAGAGAATCTTCAAAAAAGGTGGAGTGTTGCACAAATACTGGAGCAACATATGTGAACGCTA**TAG**ATGGCCAAGCCTTTGTCTCAAG	
TcPZ1-bsd-koR	KO	ACGTTAATGTGAATAATTGAGAAGAGAAAAAGTAAAGAGAGGACGGTCCAAAAACAGAATAATATATATATATATACGGCGCCATAAAAACATAATTTCTACCCCTTTTTTTTTTCTCCCTTAGCCCTCCCACACATAACCAG	
TcPZ1-puro-koF2	KO	ATAATAGTAACCATGATGGAATTGGTGAGTACAACAACTCAATGCGTCAAAAATGGCAAAAGAGAATCTTCAAAAAAGGTGGAGTGTTGCACAAATACTGGAGCAACATATGTGAACGCTA**TAG**ATGACCGAGTACAAGCCCAC	
TcPZ1-puro-koR	KO	ACGTTAATGTGAATAATTGAGAAGAGAAAAAGTAAAGAGAGGACGGTCCAAAAACAGAATAATATATATATATATACGGCGCCATAAAAACATAATTTCTACCCCTTTTTTTTTTCTCCCTCAGGCACCGGGCTTGCGGGTC	
TcPZ2-bsd-koF1	KO	TCTTTTTATGCGTGTTTACTTATTCGTGATGGTTATTGCCTTTTTTTTTTTTTTTTCGTGTTTATTTTCTTTTGGGGGATTGTTCTGTTAGTTTTTAGCATCAAATTAGGATTGGCAAAAATGGCCAAGCCTTTGTCTCAAG	
TcPZ2-bsd-koF2	KO	GCGGGCCGGTCTCCGATGGAAGATCCCATCAGACGCAGCCGCACGTCATGTATGATGGGGATATACAGAAAAGTGTGTTTGGCCGTGTTGGGTTTTGTGCGGAATGCCACCTCCATCCCCTAGATGGCCAAGCCTTTGTCTCAAG	
TcPZ2-bsd-koR	KO	TCTGGGCCGCTGACTACGGTATTTTTTGTATATATGTATATACTTACTGTATATTTTTATGTGTGTTGGCTGTGCCACGCTTCCCGCGTCAATCCTCCAACCATATGCTTGTGGCTAAATTTAGCCCTCCCACACATAACCAG	
TcPZ2-puro-koF2	KO	GCGGGCCGGTCTCCGATGGAAGATCCCATCAGACGCAGCCGCACGTCATGTATGATGGGGATATACAGAAAAGTGTGTTTGGCCGTGTTGGGTTTTGTGCGGAATGCCACCTCCATCCCC**TAG**ATGACCGAGTACAAGCCCAC	
TcPZ2-puro-koR	KO	TCTGGGCCGCTGACTACGGTATTTTTTGTATATATGTATATACTTACTGTATATTTTTATGTGTGTTGGCTGTGCCACGCTTCCCGCGTCAATCCTCCAACCATATGCTTGTGGCTAAATTCAGGCACCGGGCTTGCGGGTC	
TcPZ1gRNA-koF1	sgRNA1	GATC<u>GGATCC</u>ATCAGGGGAAAAACGGCTAGGTTTTAGAGCTAGAAATAGC	BamHI
TcPZ1gRNA-koF2	sgRNA2	GATC<u>GGATCC</u>AGACTCCCCCGGTATTGTATGTTTTAGAGCTAGAAATAGC	BamHI
TcPZ1gRNA-ckoF	sgRNA	GATC<u>GGATCC</u>GGTGGTAACTCGACTTCTTCGTTTTAGAGCTAGAAATAGC	BamHI
Com-gRNA-R	sgRNA	CAGT<u>GGATCC</u>AAAAAAGCACCGACTCGGTG	BamHI

TcPZ2gRNA-koF1	sgRNA1	GATC<u>GGATCC</u>GCGTTTGGTTCGGATTATGGGTTTTAGAGCTAGAAATAGC	BamHI
TcPZ2gRNA-koF2	sgRNA2	GATC<u>GGATCC</u>GTATGTGCGGCAGTTTATCCGTTTTAGAGCTAGAAATAGC	BamHI
TcPZ2gRNA-koF3	sgRNA3	GATC<u>GGATCC</u>AAATGGCACGCCACCAACGGGTTTTAGAGCTAGAAATAGC	BamHI
TcPZ2gRNA-ckoF	sgRNA	GATC<u>GGATCC</u>AGCCACAAGCATATGGTTGGGTTTTAGAGCTAGAAATAGC	BamHI
HX1-R	PCR	TAATTTCGCTTTCGTGCGTG	
TcPZ1-kd-F	CKO	TCAGTGGCCGCCGCGTGGATGACTACGACAGCTGCGGCAGGTTTTGTAAGGAAGTGCATACTAATCAAGATCCC	
TcPZ1-kd-R	CKO	TTTCTACCCCTTTTTTTTTCTCCCTCAAAACGGGTGGTAACTCGACTTCTTAGCCCTCCCACACATAACCAG	
TcPZ2-kd-F	CKO	CTGACGATGCCGCAAGGGAAGTGAACGTTGCCGGCCACGAAAAGAAGGAGGAAGTGCATACTAATCAAGATCCC	
TcPZ2-kd-R	CKO	CTGTATATTTTTATGTGTGTTGGCTGTGCCACGCTTCCCGCGTCAATCCTTTAGCCCTCCCACACATAACCAG	
TcPZ1-ORF-F	PCR	TTGTGTCGGACACCAATGCG	
TcPZ1-3UTR-R	PCR	ATGAGACCCGACAGACGGTTCAC	
TcPZ2-ORF-F	PCR	GGAGTGGGGATGCCTCTCTAC	
TcPZ2-3UTR-R	PCR	CTTTCAGATGCCAAATAAAGTCACTTTC	
GCaMP-F	Ca^{2+} sensor	GATC<u>TCTAGA</u>ATGGGTTCTCAT	XbaI
GCaMP-R	Ca^{2+} sensor	CTAT<u>AAGCTT</u>TCACTTCGCT	HindIII
TcPiezo2-TF	Tagging	GTCCCCGCACGCCACCCCGAGCAACCGCATGACAGAAAACCTTTCACATCGCGGCGACGCACTGAGGGGGCCCGAGGCGGGGATTGCCCCCGCTGACGATGCCGCAAGGGAAGTGAACGTTGCCGGCCACGAAAAGAAGGAGGGTACCGGGCCCCCCCTCGAG	
TcPiezo2-TR	Tagging	TTTTTTCTTCTATCCGTCTGGGCCGCTGACTACGGTATTTTTGTATATATGTATATACTTACTGTATATTTTTATGTGTGTTGGCTGTGCCACGCTTCCCGCGTCAATCCTTGGCGGCCGCTCTAGAACTAGTGGAT	

The last letter “F”s or “R”s of the primer names stand for “forward” and “reverse” primers, respectively. The underlined nucleotides indicate the introduced restriction endonuclease sites as described in the “enzyme” column. KO and CKO stands for knockout and conditional knockout, respectively. The stop codon (TAG) is introduced, as indicated in bold. bsd, blasticidin; puro, puromycin.
